# Sonocatalytic Optimization of Titanium‐Based Therapeutic Nanomedicine

**DOI:** 10.1002/advs.202301764

**Published:** 2023-07-03

**Authors:** Ruirui Zhou, Meiqi Chang, Mengjun Shen, Yang Cong, Yu Chen, Yin Wang

**Affiliations:** ^1^ Department of Ultrasound Shanghai Pulmonary Hospital School of Medicine Tongji University Shanghai 200433 P. R. China; ^2^ Laboratory Center Shanghai Municipal Hospital of Traditional Chinese Medicine Shanghai University of Traditional Chinese Medicine Shanghai 200071 P. R. China; ^3^ Materdicine Lab School of Life Sciences Shanghai University Shanghai 200444 P. R. China

**Keywords:** sonocatalytic, sonodynamic therapy, sonosensitizers, titanium‐based nanomedicine, ultrasound therapy

## Abstract

Recent considerable technological advances in ultrasound‐based treatment modality provides a magnificent prospect for scientific communities to conquer the related diseases, which is featured with remarkable tissue penetration, non‐invasive and non‐thermal characteristics. As one of the critical elements that influences treatment outcomes, titanium (Ti)‐based sonosensitizers with distinct physicochemical properties and exceptional sonodynamic efficiency have been applied extensively in the field of nanomedical applications. To date, a myriad of methodologies has been designed to manipulate the sonodynamic performance of titanium‐involved nanomedicine and further enhance the productivity of reactive oxygen species for disease treatments. In this comprehensive review, the sonocatalytic optimization of diversified Ti‐based nanoplatforms, including defect engineering, plasmon resonance modulation, heterojunction, modulating tumor microenvironment, as well as the development of synergistic therapeutic modalities is mainly focused. The state‐of‐the‐art Ti‐based nanoplatforms ranging from preparation process to the extensive medical applications are summarized and highlighted, with the goal of elaborating on future research prospects and providing a perspective on the bench‐to‐beside translation of these sonocatalytic optimization tactics. Furthermore, to spur further technological advancements in nanomedicine, the difficulties currently faced and the direction of sonocatalytic optimization of Ti‐based therapeutic nanomedicine are proposed and outlooked.

## Graphical abstract

This review mainly focuses on the sonocatalytic optimization of diversified Ti‐based nanoplatforms, including defect engineering, plasmon resonance modulation, heterojunction, modulating tumor microenvironment, as well as the development of synergistic therapeutic modalities.

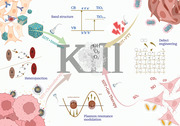

## 1 Introduction

Cancer therapy has been the theme of the modern medical research field.^[^ ^1^ ^]^ Conventional therapeutic strategies, such as radiotherapy, chemotherapy, targeted therapy, surgery, biologic therapy, etc., continuously step forward and achieve huge progress all along the way.^[^ ^2^ ^]^ Since the Feynman‐led exploration of the bottom room and the explosion of nanobiotechnology,^[^ ^3^ ^]^ therapeutic routes have been prompted in a revolutionary direction. Abundant nanomedicine platforms with versatile biological effects, including photothermal therapy (PTT), photodynamic therapy (PDT), chemodynamic therapy (CDT), sonodynamic therapy (SDT), and other therapeutic modalities, attract tremendous attention from the scientific communities, blooming the development of the disease‐ and patient‐specific diagnosis and treatment. These pioneering studies integrate internal and/or external energy‐triggered nanobiotechnology into traditional therapeutic strategies and have emerged as a research hotspot in the field of oncology. However, some obstacles remain existed in the further biomedical applications and mass production. For example, photodynamic‐related treatment destroys tumor cells by generating reactive oxygen species (ROS) after activating the photosensitizers by light of a certain wavelength,^[^ ^4^ ^]^ which shows excellent specificity and selectivity^[^ ^5^ ^]^ due to the concentration characteristics of photosensitizer at tumor site. However, the nonuniformity of microstructure will lead to the loss of directionality of beam propagation.^[^ ^6^ ^]^ The limited tissue penetration feature is responsible for the inefficient utilization of PDT for the treatment of deep‐seated tumor.^[^ ^7^ ^]^

With a similar operating principle to PDT, sonocatalytic‐based treatment with non‐invasive, controllable and low‐cost characteristics is a distinct therapeutic modality that employs ultrasound (US) power to activate sonosensitizers,^[^ ^8^ ^]^ which has been extensively used in treating deep‐seated lesions for several years.^[^ ^9^ ^]^ As one of the main elements in SDT, US can penetrate significantly deeper areas of biological tissue because of its non‐radiative properties and low tissue attenuation coefficient, which can increase the cell membrane permeability and enhance the cellular uptake of sonosensitizers.^[^ ^10^ ^]^ Moreover, sonosensitizer acts as the other critical parts that specifically kills tumor cells through apoptosis and/or necrosis and influences SDT efficacy.^[^ ^11^ ^]^ In general, there are two main types of sonosensitizers, including organic and inorganic sonosensitizers.^[^ ^10^ ^]^ The organic sonosensitizers comprise of xanthene compounds,^[^ ^12^ ^]^ porphyrins and phthalocyanines,^[^ ^13^ ^]^ fluoroquinolone antibiotics,^[^ ^14^ ^]^ phenothiazine compounds,^[^ ^15^ ^]^ natural products,^[^ ^16^ ^]^ etc., which are characterized by clear chemical structure, well‐managed production process and easy monitoring.^[^ ^17^ ^]^ However, the undesirable phototoxicity, poor water solubility/low bioavailability, as well as low tumor tissue‐targeting capability hinder their clinical application.^[^ ^10^ ^]^ By contrast, the inorganic bio‐nanosystems such as Fe_3_O_4_, SiO_2_, MnO_2_, and TiO_2_ have become the focus of current studies due to their excellent biocompatibility and high stability for in vivo translation.^[^ ^18^ ^]^ Among them, Ti‐based nanomaterials have been employed to construct diverse functional nanosystems due to the distinct structural and remarkable physicochemical features. For example, the semiconductor TiO_2_ is a typical sonosensitizer with a variety of beneficial features, including easy fabrication, chemical inertness in biological systems, low phototoxicity and high stability under physiological conditions.^[^ ^19^ ^]^ However, the pure Ti‐based sonosensitizers suffer from the low quantum yield and poor sonodynamic effect due to the fast recombination of the activated electrons and holes,^[^ ^20^ ^]^ necessitating the development of alternative and optimization techniques to compensate for these drawbacks.

**Scheme 1:**
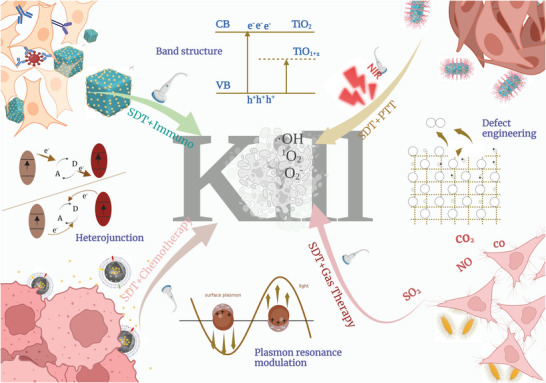
Schematic illustration of sonocatalytic optimization of titanium‐involved therapeutic nanomedicine, including synthesis guidance and optimization strategies.

In this review, we mainly focus on the optimization of Ti‐based nanosonosensitizers in sonocatalytic therapeutic nanomedicine, highlight the very‐recent optimized sonoplatforms at nanoscale, and systematically summarize/discuss the dominant fabrication strategies, surface modification/functionalization approaches and augmented performances in disease therapeutics (**Scheme** **1**). Especially, the facing challenges and potential prospects of Ti‐based nanoplatforms for subsequent clinical translations and commercialization are discussed and outlooked.

## 2 Classification and Synthesis Guidance for Titanium‐Based Nanomedicine

Sophisticated nanostructures endowed with specific properties arise from different synthesis strategies. We compiled seven leading preparation methods for the fabrication of Ti‐based nanoparticles and biomaterials, including liquid exfoliation, sol–gel process, chemical vapor deposition (CVD), templating method, and solvothermal/hydrothermal approach from plenty of historical studies. Below are descriptions of each synthesis technique along with the representative examples.

### 2.1 Classification

Titanium‐based nanomedicine can be categorized into the following groups according to their biomedical applications, such as titanium oxides (e.g., TiO_2_, TiO_1−x_), titanium sulfides (e.g., TiS_2_), titanium nitrides (e.g., TiN), titanium hydrides (e.g., TiH_1.924_), titanium carbides (e.g., Ti_2_C and Ti_3_C_2_ MXenes), Ti‐based metal‐organic framework (MOF), titanium nanocomplex (e.g., BaTiO_3_, TiP), and their nanocomposites (e.g., Ti_3_C_2_@TiO_2−x_, Ti_3_C_2_/CuO_2_@BSA, MnO_x_/Ti_3_C_2_).

### 2.2 Top‐Down Approach

#### 2.2.1 Liquid Exfoliation

**Figure 1:**
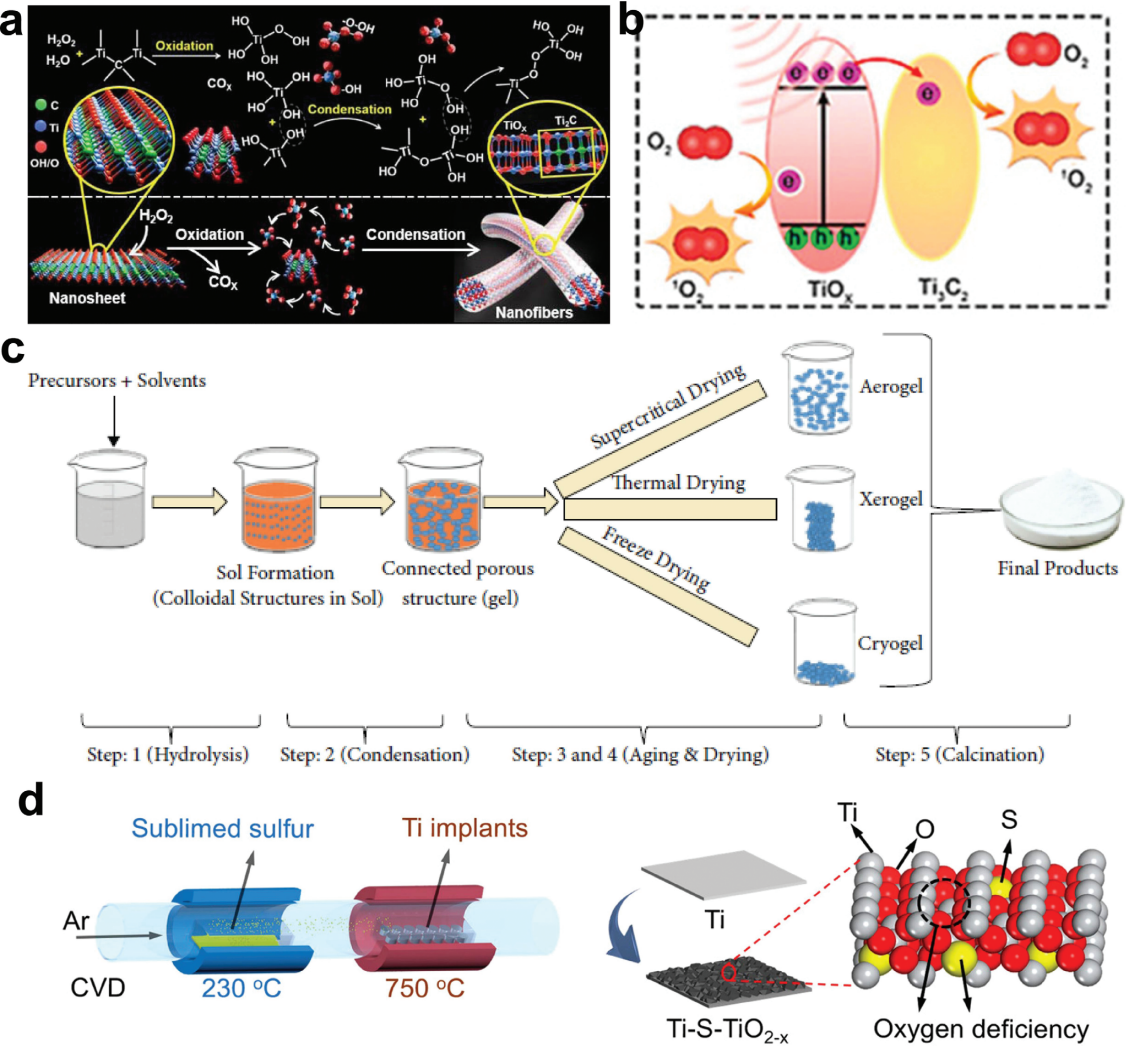
Synthesis methods for titanium‐based sonosensitizers. a) Mechanism of the H_2_O_2_‐rich environment triggering the synthesis of nanofibers from Ti_2_C(OH)_X_ nanosheets. Reproduced with permission.^[^ ^25^ ^]^ Copyright 2022, American Chemical Society. b) ROS generation process under US irradiation: the defect structure encourages the transformation of charge carriers and the capture of the electron, restraining the recombination of the electron and hole. Reproduced with permission.^[^ ^26^ ^]^ Copyright 2022, Elsevier. c) Synthetic route of sol–gel strategy. Reproduced with permission.^[^ ^27^ ^]^ Copyright 2022, Taylor & Francis Inc. d) The synthesized Ti‐S‐TiO_2−x_ via CVD method. Reproduced with permission.^[^ ^28^ ^]^ Copyright 2020, American Chemical Society.

Exfoliation is the most commonly used top‐down method for preparing two‐dimensional materials, such as graphene, layered double hydroxides, black phosphorus (BP) as well as the family of MXenes.^[^ ^21^ ^]^ Layered crystals can be exfoliated into nanosheets due to their strong chemical bonds in‐plane but weak out‐of‐plane, van der Waals bonds, which leads to remarkable crystal surface area for versatile applications.^[^ ^22^ ^]^ Typically, MXenes are created by exfoliating MAX phases in hydrofluoric acid (HF)^[^ ^23^ ^]^ and developed various crystal compounds comprising Ti_2_C, Ta_4_C_3_, Ti_3_CN, etc. To date, sophisticated 2D Ti‐based MXene nanosensitizers for augmenting SDT efficacy are rationally designed. For example, our group fabricated Ti_3_C_2_ MXene nanosheets by using HF etching and utilized them as a platform to modify CuO_2_ nanodots as well as bovine serum albumin (BSA) on the surface.^[^ ^24^ ^]^ The as‐synthesized Ti_3_C_2_/CuO_2_@BSA nanosheets showed excellent stability and desirable electrical conductivity. Similarly, You et al. successfully prepared Ti_2_C nanosheets by immersing Ti_2_AlC into 30% HF and stirring for 72 h at 50 °C.^[^ ^25^ ^]^ After the surface modification, the obtained Ti_2_C(OH)_X_ nanosheets manifest long blood circulation with nanofiber structure (**Figure** **1**a,b).

Typically, liquid exfoliation involves two phases. Intercalation is the initial step to enhance layer separation and decrease adhesion between layers. To disrupt the weak van der Waals force between neighboring layers, the second step is sonication.^[^ ^29^ ^]^ For example, Cheng et al. facilely fabricated TiN nanodots by utilizing probe and bath sonication liquid phase exfoliation. The as‐prepared TiN showed dot‐like morphology with an average diameter of 1.54 ± 0.53 nm.^[^ ^30^ ^]^ Subsequently, this technique was used by Liu and his co‐workers to create TiH_1.924_ nanodots from its powder form.^[^ ^31^ ^]^ As molecular entities distributed in a solvent, nanosheets can be aggregated or formed into a variety of structured nanostructures. They can also be combined with other molecules to form lamellar nanocomposites (NCs).^[^ ^32^ ^]^ The exfoliation efficiency can be influenced by solvent properties, solvent transformations, sonication parameters, etc.^[^ ^33^ ^]^ However, gentler approaches to avoiding or reducing the frequency of use of HF are urgently needed due to its general high toxicity of HF.

### 2.3 Bottom‐Up Fabrication

#### 2.3.1 Sol–Gel Method

As a wet chemical method, sol–gel processing is particularly useful in fabricating metal oxides and organic–inorganic hybrid materials. Typical sol–gel processing contains hydrolysis and condensation reactions and the size of productions ranging from 1 to 100 nm, which can be manipulated by altering the concentration and aging time (Figure 1c). The charge of nanoparticles causes them to stabilize electrostatically, preventing particle collision.^[^ ^34^ ^]^ This method is frequently used to create crystalline or amorphous structures with exceptional uniformity and cleanliness.^[^ ^35^ ^]^ For example, Wei et al. prepared TiO_2_‐based sonocatalytic nanoagents (SCN) via a sol–gel method.^[^ ^36^ ^]^ Firstly, they mixed tetrabutyl titanate, ethanol, and ultrapure water and kept continuously stirring at 30 °C, then added DSPE‐PEG2000‐NH_2_, B16F10 cell membranes to the aforementioned mixtures, the SCN@B16F10M/PEG‐aPD‐L1 was finally obtained after the conjugation of PD‐L1. More specifically, Zuo et al. synthesized mesoporous titanium dioxide nanoparticles (mTNPs) employing the template cetrimonium bromide and the precursor titanium isopropylate and then successfully loaded L‐Arg onto the surface.^[^ ^32^ ^]^ Transmission electron microscopy (TEM) image showed that the mTNPs had a consistent spherical morphology with a diameter of about 50 nm. The sol–gel method features low reaction temperature, simple and easy operation of equipment, good process repeatability, and can effectively inhibit the growth and coagulation process of particles. As a result, the product is homogeneous in size and has appropriate dispersion.

#### 2.3.2 Chemical Vapor Deposition (CVD)

Vapor deposition, typically carried out in a vacuum chamber, refers to particular process in which vapor‐phase materials are condensed to form materials in a solid phase. The procedure is known as chemical vapor deposition because it involves related chemical reactions. To be more precise, thermal energy dries the deposition process and warms the air in the coat chamber.^[^ ^37^ ^]^ Several Ti‐based nanoparticles have been created in desirable single crystals via this method.^[^ ^38^ ^]^ The CVD growing technique is flexible and can be utilized as an universal method for creating a wide range of high‐quality 2D ultrathin transition metal carbides crystals.^[^ ^39^ ^]^ For example, Wu et al. ingeniously synthesized Ti‐S‐TiO_2−x_ in a tube furnace where Ti implant immersed in an atmosphere of argon and sulfur.^[^ ^28^ ^]^ The S‐doped Ti implant with oxygen deficiency structures features exceptionally powerful antibacterial performance as well as enhanced photothermal and sonodynamic treatment efficacy (Figure 1d). Another paradigm reported by Wang et al. introduced oxygen defects onto the BaTiO_3_ via the CVD method, endowing the as‐obtained Bi‐doped oxygen‐deficient BaTiO_3_ with higher ROS production efficiency.^[^ ^40^ ^]^ CVD benefits the regulated nanoparticle features, including crystal structure, excellent film durability, straightforward scale‐up, and controlled surface morphology. However, this technique has also been claimed to face several obstacles, including the difficulty in deposition of multicomponent materials and the potential for chemical dangers owing to the usage of corrosive, poisonous, and explosive precursor gases.^[^ ^27^ ^]^

#### 2.3.3 Solvothermal/Hydrothermal Method

Solvothermal/hydrothermal method are both widely used in fabricating TiO_2_ nanoparticles (NPs), nanorods, nanowires, etc. Hydrothermal processing is the dissolution and recrystallization of materials using heterogeneous reactions with aqueous solvents or mineralizers under high pressure and temperature conditions. The hydrothermal approach offers exclusive benefits for the fine synthesis of nanomaterials given the tightly regulated diffusivity in a closed system, which favors a decrease in particle agglomeration, narrows the size distribution of the particles, as well as regulates the particles morphology.^[^ ^41^ ^]^ As a typical example in the field of SDT, Li et al. developed a Cu_2−x_O‐BaTiO_3_ piezoelectric heterostructure by sealing barium hydroxide octahydrate, anatase titanium dioxide and water in an autoclave and kept at 200 °C for 72 h.^[^ ^42^ ^]^ In another study above‐mentioned, Lin et al. created Ti_3_C_2_@TiO_2−x_ nanoplatform by allowing TiO_2_ NPs to hydrothermally grow in situ on the 2D Ti_3_C_2_ nanosheets.^[^ ^24^ ^]^ Due to the use of organic solvents with high boiling points, the solvothermal approach differs from the hydrothermal method in that it allows the reaction temperature to reach a higher level.^[^ ^37^ ^]^ The internal pressure generated by the autoclave is largely influenced by the temperature and volume of solution.^[^ ^43^ ^]^ Our group first produced curb barium titanate (BTO) by a solvothermal method utilizing the precursors Ba(OH)_2_·H_2_O and Ti[O(CH_2_)_3_CH_3_]_4_, and then they undertook a simple thermal anneal to convert curb BTO into tetragonal BTO.^[^ ^44^ ^]^ The as‐designed piezoelectric materials integrated with US response characteristics and could efficiently eliminate tumors due to ROS generation ability. Notably, the solvothermal approach typically has better control over the size, shape distributions, and crystallinity of nanoparticles than hydrothermal methods, exhibits tremendous potential for fabricating exquisite nanoplatforms in future anti‐cancer applications.^[^ ^43^ ^]^

#### 2.3.4 Soft and Hard Templating Methods

Classical templating techniques, including soft and hard templates, are generally utilized in fabricating mesoporous materials, such as clay minerals, metal‐organic framework (MOF), and materials based on metal oxides. Due to their adjustable pore structure, high porosity, and enormous surface area, mesoporous materials can be widely used in drug delivery systems.^[^ ^45^ ^]^ Hard‐templating synthesis, also named nano‐casting, relies on solid materials as a “model” to replicate various nanomaterials.^[^ ^46^ ^]^ This procedure can avoid the effects of hydrolysis and polycondensation rates of Ti precursors and co‐assembly with surfactants.^[^ ^47^ ^]^ The size and shape of the sample particle are directly influenced by the stable structure of rigid material, which is known as a “hard template.” There is a large selection of hard templates, including porous anodic aluminum oxide, plastic foam, polymer microspheres, silica and porous membranes.^[^ ^48^ ^]^ As a typical example, polystyrene sphere as the hard‐template was applied for the preparation of hollow TiO_2_ through calcination process. This method makes it possible to precisely manage the size and specifications of products.^[^ ^48b^ ^]^ Using colloidal silica spheres as the hard template, cancer cell membrane‐coated C‐TiO_2_ hollow nanoshells have been prepared for combined sonodynamic and hypoxia‐activated chemotherapy. The special cavity structure of C‐TiO_2_ hollow nanoshells could achieve the loading of tirapazamine.^[^ ^49^ ^]^ Similarly, collagenase‐loaded hollow TiO_2_ nanoparticles have been constructed by Luo et al. for US imaging‐guided SDT in a pancreatic carcinoma xenograft model through etching the SiO_2_ template.^[^ ^50^ ^]^ Contrarily, the synthesis of soft‐templating requires flexible surfactants that have the ability to self‐assemble into homogenous micelles. Moreover, spontaneous micellization can trigger the coassembly of amphiphilic surfactants and inorganic precursors through noncovalent bonds. By adjusting the molecular weights, length of the hydrophilic/hydrophobic chains, and reactant ratio, it is possible to get over the limitations of hard‐templating synthesis and conveniently tailor the pore characteristics.^[^ ^51^ ^]^ Mesoporous TiO_2_ was originally created via the soft‐templating method with titanium isopropoxide bisacetylacetonate serving as the titanium supply and alkyl phosphate surfactants as the structure‐directing agent.^[^ ^52^ ^]^ On the basis of that, several strategies have developed to improve the thermally stability of mesoporous TiO_2_.^[^ ^53^ ^]^ Chen et al. developed a versatile soft‐templating strategy to produce mesoporous anatase TiO_2_ nanoparticles (MTNs) with a nanosized single‐crystalline structure.^[^ ^54^ ^]^ Between the hydrolyzed by‐product of the titanium source (tetrabutyl titanate) and acetic acid, this organic/inorganic self‐assembly process in situ self‐generates the micelle (as the soft‐template). Using a modified soft‐templating technique, Hu et al. created mTiO_2_‐PPy NCs by fusing the DOX and aspirin prodrugs (P‐DOX and P‐Aspirin), which coupled aspirin's anti‐inflammatory properties with SDT for antitumor therapy.^[^ ^55^ ^]^ The desirable monodispersity and well‐defined mesoporous characteristics provide the novel sonosensitizers with superb optoelectronic device performance for biomedical applications.

#### 2.3.5 High‐Temperature Pyrolysis Strategy

High‐temperature pyrolysis is a bottom‐up approach to obtain high‐performance nanomaterials through the thermal decomposition of select precursors at appropriate pyrolysis temperatures, which have been widely used due to their unique advantages, including their excellent crystallinity, monodispersity, as well as high dispersibility in organic solvents. As the most representative process, the thermal decomposition reactions of metal‐surfactant complexes and organometallic compounds were performed in the presence of surfactants under high‐temperature environments.^[^ ^56^ ^]^ By using a standard organic‐phase synthesis technique, Cheng et al. generated high‐quality TiO_1+x_ nanorods through a seed‐mediated process.^[^ ^57^ ^]^ In this procedure, TiCl_4_ was injected into oleylamine and 1‐octadecene and heated to 320 °C. Moreover, their group fabricated Ti_3_C_2_ MXene nanosheets (Ti_3_C_2_ NSs) using the same strategy, Ti_3_C_2_ NSs with increased oxygen defect greatly improved the SDT efficiency.^[^ ^26^ ^]^ Li's group hybridized TiN nanoparticles with Fe and N co‐doped carbon NSs.^[^ ^58^ ^]^ During high‐temperature pyrolysis, Fe‐doped carbon nitride NSs can be decomposed to generate Fe‐N co‐doped carbon NSs, and TiO_2_ nanoparticles were converted into TiN nanoparticles during the high‐temperature nitriding process. In short, this simplicity of preparation and low cost of this method make it popular for producing Ti‐based NMs with customizable form and composition.^[^ ^29^, ^59^ ^]^

## 3 Optimization Strategy for Enhanced Therapeutic Efficacy of SDT

### 3.1 Surface Modification of Ti‐Based Nanosonosensitizers

**Figure 2:**
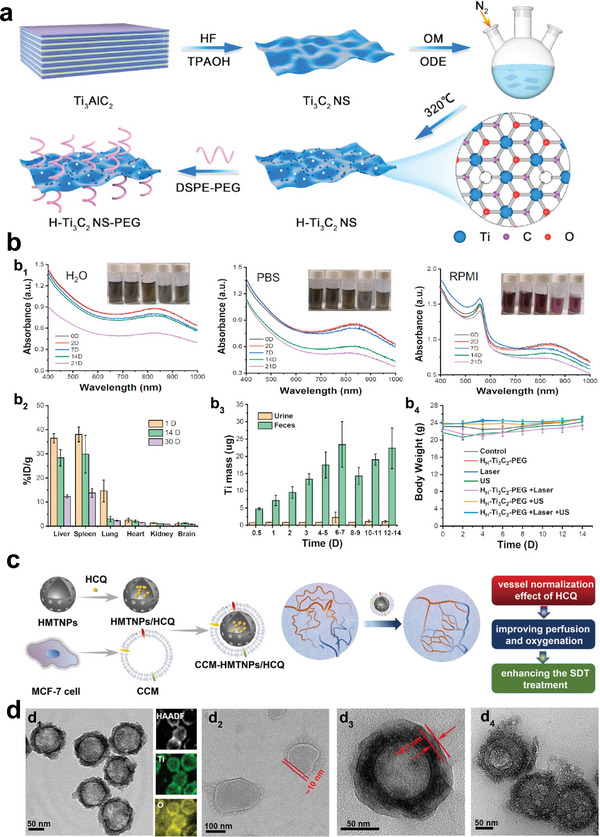
Surface modification of Ti‐based sonosensitizers. a) H‐Ti_3_C_2_ nanosheets as fabricated through an etching and chemical exfoliation method and modified by DSPE‐PEG. b‐b_1_) UV–vis–NIR spectra of H_H_‐Ti_3_C_2_‐PEG nanosheets in H_2_O, PBS, and RPMI, respectively; b_2_) In vivo distribution profile of H_H_‐Ti_3_C_2_‐PEG nanosheets; b_3_) The detected Ti mass in urine and feces; b_4_) The body weight change of mice after different treatments. Reproduced with permission.^[^ ^26^ ^]^ Copyright 2022, Elsevier. c) Synthetic route of CCM‐HMTNPs/HCQ and vessel normalization effect of HCQ. d‐d_1_) TEM image and the correspondent high‐angle annular dark‐field scanning TEM‐based elemental mapping image of hollow mesoporous titanium dioxide nanoparticles (HMTNPs), d_2_) TEM images of CCM, d_3_) CCMHMTNPs/HCQ and d_4_) CCM‐HMTNPs/HCQ, respectively. Reproduced with permission.^[^ ^70^ ^]^ Copyright 2019, American Chemical Society.

Ti‐based nanoparticles have the property of uneven dispersion due to their intrinsic instability.^[^ ^60^ ^]^ To solve this problem, several strategies of surface modification have been formulated. Polyethylene glycol (PEG)^[^ ^61^ ^]^/polyvinylpyrrolidone (PVP)^[^ ^62^ ^]^/hyaluronic acid (HA)^[^ ^63^ ^]^/carboxymethyl dextran^[^ ^19b^ ^]^/polyetherimide^[^ ^64^ ^]^/polypyrrole (PPy)^[^ ^65^ ^]^ polymers could lessen the adherence of serum proteins to nanomaterials as well as the removal of nanomaterials (NMs) by the reticuloendothelial system. Among them, PEGylation is the most common approach. For example, Cheng et al. designed Ti_3_C_2_ MXene nanosheets via a two‐step method.^[^ ^26^ ^]^ H‐Ti_3_C_2_‐PEG NSs exhibited excellent stability and biocompatibility after being modified with PEG, reflected in both in vivo and in vitro studies (**Figure** **2**a,b). On the other hand, NMs build up in sufficient numbers at the tumor locations as a result of the modification of targeting molecules. For example, the folic acid (FA)‐coated NMs could target and activate folate receptors;^[^ ^64^, ^66^ ^]^ triphenylphosphine‐modified NMs could recognize mitochondria;^[^ ^67^ ^]^ biological membranes (mesenchymal stem cells,^[^ ^68^ ^]^ red blood cells,^[^ ^69^ ^]^ cancer cells,^[^ ^70^ ^]^ etc.), which can endow NMs with unique biocompatible and versatile functions (Figure 2c,d). To increase the selectivity and efficacy of photoexcited TiO_2_ in killing cancer cells, Jiang et al. combined TiO_2_ NPs with a monoclonal antibody.^[^ ^71^ ^]^ TiO_2_ NPs could accumulate on cancer cells due to the specific response between the antibody in antibody‐TiO_2_ conjugates and the antigen on cancer cells. Similarly, Tijana Rajh and colleagues fabricated TiO_2_ NPs covalently linked to an antibody through a dihydroxybenzene bivalent linker to selectively recognize glioblastoma multiforme.^[^ ^72^ ^]^ Cao et al. designed a mitochondria‐targeted triphenylphosphine (TPP) and AS1411 aptamer‐modified Au‐TiO_2_ (Au‐TiO_2_‐A‐TPP) to enhance the effectiveness of organ‐targeted computed tomography imaging and SDT.^[^ ^67a^ ^]^ The Au‐TiO_2_‐A‐TPP manifested high tumor accumulation as well as low systematic side effects. As another representative example, Nobuaki et al. studied the preparation of protein‐immobilized TiO_2_ NPs and their SDT efficiency in vitro and in vivo.^[^ ^73^ ^]^ HepG2 cells were used to study the uptake behavior of TiO_2_ NPs treated with pre‐S1/S2 (a model protein recognized by hepatocytes). It took 6 h for the cells to sufficiently uptake the TiO_2_ NPs. The effect of the TiO_2_/US treatment on HepG2 cell growth confirmed the role of model proteins.

To sum up, diversiform targeting units with multifunctional functionalities have so far been investigated. Therefore, researchers need prudently select the modified strategies based on the physicochemical properties of titanium‐involved nanomaterials.

### 3.2 Defect Engineering of Ti‐Based Nanosonosensitizers

Owing to the shortcomings of limited light‐sensitive range (only in the UV scope of irradiation) and the recombination of charge carriers among Ti‐based nanomaterials, various methods such as introducing external species or modifier/dopants have been of great interest. For instance, N‐, F‐, P‐, C‐, and Ce‐doped TiO_2_ nanomaterials exhibited the excellent application performance due to the changes in band gap and electron–hole separation efficiency.^[^ ^74^ ^]^ As a typical example, Kang et al. prepared N‐doped oxygen defective N/TiO_2−x_ mesocrystal nanocubes for enhanced photodegradation. N‐doping and oxygen vacancies can effectively increase the visible light adsorption and promote the separation of photo‐generated charge carriers.^[^ ^74d^ ^]^ The discovery that the recombination of e–h in defects or on surface of defects has drawn significant attention.^[^ ^75^ ^]^ The introduction of oxygen vacancy can influence the charge transport and the electron–hole recombination process. Herein, several oxygen defects‐related nanoplatforms have been explored recently, significantly promoting the efficiency of sonodynamic anti‐tumor therapy.

Defect engineering can improve the efficiency of Ti‐based sonosensitizer by altering charge transfer and suppressing electron–hole recombination. It can be divided into two categories: anion vacancies and cation vacancies. The most prevalent anion vacancies, oxygen vacancies, have a low formation energy and are more prevalent in transition‐metal oxides. In addition, cation vacancies significantly moderate the electronic structure and physicochemical properties of metal compounds due to various electron configurations and orbital distributions.^[^ ^76^ ^]^ Numerous catalytic materials with metal cation vacancies have been created, such as titanium vacancies in TiO_2_.^[^ ^77^ ^]^ Furthermore, the inherent features of the materials, such as microstructure, electronic structure, atom coordination number, carrier concentration, or electrical conductivity, can be changed by engineering specific defects.^[^ ^78^ ^]^ As a typical paradigm, Kong et al. introduced defects of O vacancy and Ti^3+^ in surface and bulk TiO_2_ nanosheets through the ambient‐temperature plasma engraving treatment. Then, the band gap of the 2D TiO_2_ nanosheets decreased from ≈3.13 to 2.88 eV and more active sites of material surface emerged, which is attributed to the doping defect of O vacancy and Ti^3+^.^[^ ^79^ ^]^

As a typical paradigm, titanium monoxide (TiO_1+x_) nanorods with horseradish‐peroxidase‐like enzyme activity had been reported by Cheng et al.^[^ ^57^ ^]^ As‐synthesis TiO_1+x_ containing Ti^2+^, Ti^3+^, and a trace quantity of Ti^4+^, as shown by several banding energy peaks in X‐ray photoelectron spectroscopy, became the solid evidence of the presence of oxygen‐deficient structure. TiO_1+x_ ultrafine nanorods fabricated by a high‐temperature organic strategy grow in a seed‐mediated mechanism and show uniform morphology and size. TiO_1+x_ NRs are more capable of producing ROS than TiO_1+x_/TiO_2_ nanocomplexes and commercial TiO_2_ NPs, because the O_2_‐deficient structure can limit the recombination of electron–hole pairs. The Pt‐TiO_2_ heterostructure created by Lin and his colleagues exhibited high chemo‐sonodynamic synergistic efficacy and oxygen generation ability due to the presence of Pt, ultimately alleviating tumor hypoxia.^[^ ^80^ ^]^

**Figure 3:**
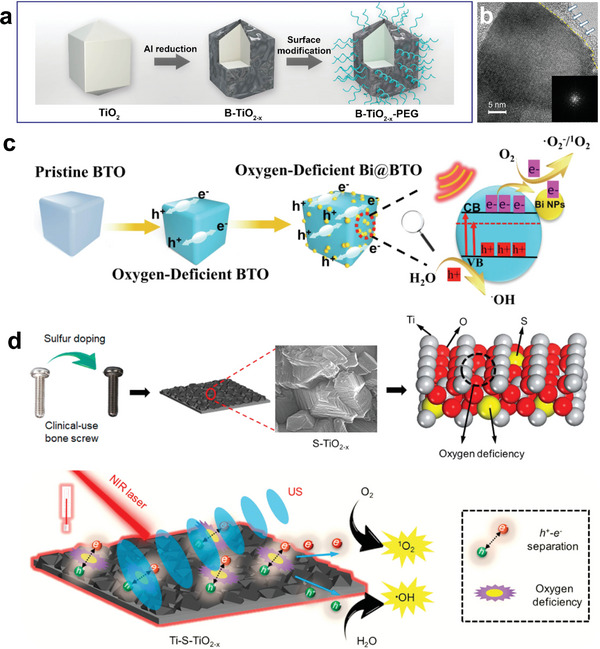
Defect engineering of Ti‐based sonosensitizers. a) Diagram of the aluminum reduction process used to fabricate B‐TiO_2−x_. b) High‐resolution TEM images of oxygen‐deficient amorphous shell of B‐TiO_2−_ *_x_* and a single‐crystalline core. Reproduced with permission.^[^ ^61a^ ^]^ Copyright 2018, American Chemical Society. c) Synthesis procedure of Bi‐doped oxygen‐deficient BaTiO_3_. Reproduced with permission.^[^ ^40^ ^]^ Copyright 2022, Elsevier. d) Enhancing sonocatalytic‐photothermal efficiency by defect engineering in sulfur‐doped bone screws. Reproduced with permission.^[^ ^28^ ^]^ Copyright 2020, American Chemical Society.

Our team coated the surface of TiO_2_ nanocrystals with an oxygen‐deficient TiO_2–x_ layer using a simple aluminum‐reduction technique.^[^ ^61a^ ^]^ The representative core‐shell structure with an apparent oxygen‐deficient amorphous shell endows B‐TiO_2–x_ NPs with high photothermal‐conversion and SDT efficiency (**Figure** **3**a,b). Ma and his colleagues successfully constructed black oxygen‐deficient TiO_2_ (B‐TiO_2_) nanoplatforms for synergistic PTT/SDT.^[^ ^81^ ^]^ Similarly, Bi doped oxygen‐deficient BaTiO_3_ nanoparticles had been created by Wang and his co‐workers, achieving high SDT efficiency for deep ovarian cancer therapy^[^ ^40^ ^]^ (Figure 3c). The piezoelectric material BaTiO_3_ can create a dynamic internal electric field when subjected to US, which facilitates continual separation of electrons and holes.^[^ ^44^ ^]^ After defect engineering, oxygen‐deficient BaTiO_3_ exhibited a cubic shape and flawed edges, and the signal of oxygen defect was observed by electron paramagnetic resonance. Furthermore, the local piezoelectric response was demonstrated by the varying amplitude of the butterfly‐shaped loop. Bi‐doped oxygen‐deficient BaTiO_3_ showed a strong sono‐current intensity, indicating that charge transfer was boosted at the BaTiO_3_ and Bi hetero‐interface.

Oxygen‐deficient TiO_2_ has also been explored for antimicrobial and biomedical therapy. A sulfur‐doping Ti‐based NPs created by Wu et al. reached 99.995% antibacterial efficiency against *Staphylococcus aureus* under near‐infrared light and US treatments^[^ ^28^ ^]^ (Figure 3d). The cutting‐edge Ti‐based implant displayed safer and more controlled antibacterial performance as compared to previous antibacterial approaches, significantly expediting osseointegration.

On this ground, defect engineering may lead to advancements in the fundamental electro‐catalysis and photocatalysis capabilities of nanomaterials as well as new suggestions for the thoughtful design of NMs.^[^ ^82^ ^]^ Many oxygen‐deficient nanoplatforms have been created for cancer therapies, and this approach not only helps SDT but also other medicines.^[^ ^83^ ^]^ However, studies on Ti‐based sonosensitizers are still a dearth in the field of SDT. More durable performance might be seen when metal‐oxygen clusters with Ti^3+^ (like MOF) are coupled with organic and inorganic hybrid components.

### 3.3 Plasmon Resonance Modulation of Ti‐Based Nanosonosensitizers

**Figure 4:**
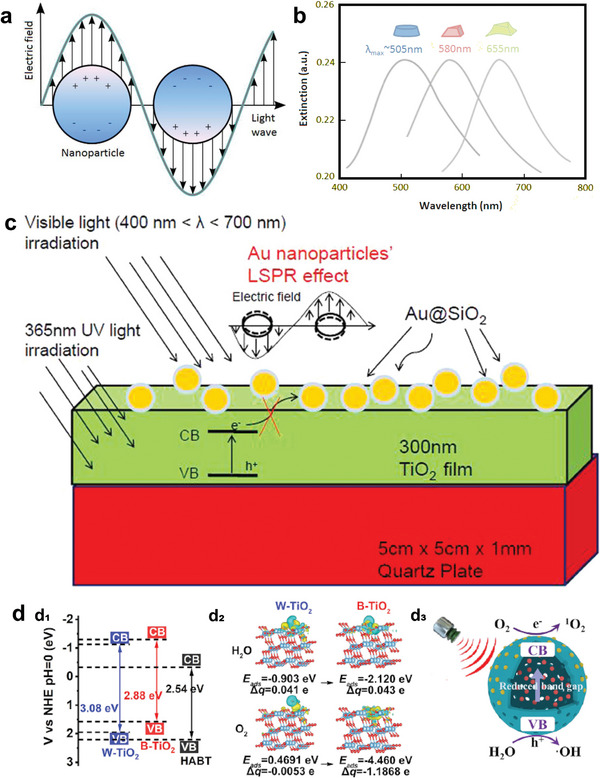
Plasmon resonance modulation of Ti‐based sonosensitizers. a) The induced plasmons oscillate locally to the nanostructure in LSPR as opposed to along the metal‐dielectric contact, which is how it differs from SPR. b) Nanostructure shape affects the maximum of extinction wavelength (*λ*_max_). Reproduced with permission.^[^ ^85^ ^]^ Copyright 2014, MDPI. c) The scheme of the LSPR effect of Au nanoparticles on the photocatalytic activity of TiO_2_ film.^[^ ^88^ ^]^ d‐d_1_) Band structures of W‐TiO_2_, B‐TiO_2_, hollow black TiO_2_ nanosphere (HABT); d_2_) Charge difference distribution of optimized adsorption and the calculated Bader charge of H_2_O molecule and O_2_ molecules on W‐TiO_2_ and B‐TiO_2_; d_3_) The proposed mechanism of HABT for enhanced SDT. Reproduced with permission.^[^ ^89^ ^]^ Copyright 2022, American Chemical Society.

Metallic nanoparticles in colorful colloidal fluids have long drawn the attention of scientists. This phenomenon, indeed the tiny particle effect, was known as surface plasmon resonances. The coherent oscillation of conduction band electrons caused by the interacting electromagnetic field is the physical cause of light absorption.^[^ ^84^ ^]^ According to this concept, plasmonic metal nanoparticles (Au, Ag, Pt, Fe, etc.) have been coupled with a bound of dielectric oxide materials such as SiO_2_, TiO_2_, and ZrO_2_ to establish metal‐oxide hybrid nanoplatforms.^[^ ^84^ ^]^ This phenomenon is referred to as localized‐surface‐plasmon‐resonance (LSPR) when the frequency of the incident electromagnetic wave is similar to or equal to the plasmon frequency. Some structural elements, including dimension, form, as well as spacing, have a significant impact on LSPR.^[^ ^85^ ^]^ Hot electrons are created when a semiconductor material's surface interacts with a high‐energy photon source (**Figure** **4**a,b). These electrons are able to leave the surface and go to the nearby substance.^[^ ^86^ ^]^ Methodologies have been developed to engineer LSPR qualities since the impact of LSPR depends on the size, shape, and regional dielectric environment of the material.^[^ ^87^ ^]^

As the electron‐trapping characteristics of metals provide reduction processes with an active site on the surface of photocatalytic particles, this noble metal‐coupling technique is not only capable of improving the photocatalytic property of TiO_2_ nanoparticles;^[^ ^75^ ^]^ furthermore, it can also augment the effect of SDT by virtue of enhancing the separation and suppressing the recombination of e^−^/h^+^ pairs.^[^ ^90^ ^]^ Jae Hyung Park and his co‐works, for the first time, utilized this strategy in SDT treatment to increase the production of ROS by combining Au with TiO_2_ NCs.^[^ ^91^ ^]^ Noble metal Au, in this procedure, interacts with TiO_2_ and prolongs the lifetime of electrons, leading to fivefold the ROS yield compared with pure TiO_2_. After that, Au@TiO_2_,^[^ ^92^ ^]^ Fe‐TiO_2_,^[^ ^61b^ ^]^ PT‐TiO_2_^[^ ^80^ ^]^ were successfully designed and exhibited excellent generation ability of both ^1^O_2_ and •OH in comparison with pure TiO_2_. It is widely accepted that TiO_2_‐based nanomaterials inherently feature fast electron–hole pair recombination rate and wide bandgap (3.2 eV), which strongly limit the ROS generation under US stimulation.^[^ ^93^ ^]^

High plasmonic response and advanced colloidal assembly technology have been the two main elements influencing the frequent use of Au and Ag in the field of plasmonics for many years.^[^ ^94^ ^]^ However, in addition to resonance regions that are spectrally mismatched, metals typically suffer from high losses from interband transitions that are spectrally located quite close to the resonance areas. Moreover, metals like Au experience chemical instabilities despite having powerful plasmonic responses. Strategies such as using thick encapsulating silica shells improve stabilities to some extent but decrease the application performance. Herein, alternative plasmonic materials have been explored for superior chemical characteristics in SDT.

Liu et al. modified Au and CDs on hollow black TiO_2_, and the products showed a spherical hollow structure with diameter of 100–200 nm and revealed a significant signal of ROS generation under US, due to the presence of O_v_ and Ti^3+^ in the B‐TiO_2_ as well as the LSPR of Au NPs^[^ ^89^ ^]^ (Figure 4d). Notably, the triple‐enzyme mimetic activities of the above‐mentioned nanoplatform allowed it to alleviate hypoxia in the TME and inhibited the expression of immunosuppressive mediators, providing a prospective strategy for enhancing anti‐tumor SDT efficiency. Surface plasmon affect semiconductor photocatalyst in a number of ways because of the catalytic ability of noble metals and quantum tunneling effect.^[^ ^84^ ^]^

In summary, plasmon resonance modulation is a technique for controlling e^−^/h^+^ pairs that couples noble metals to nanomaterials. These metal‐oxide nanoplatforms own electron‐trapping properties, thus providing nanosensitizers with photo‐ and sonodynamic abilities.^[^ ^95^ ^]^ According to studies, Au@SiO_2_/TiO_2_ has a greater LSPR than Au/TiO_2_ and has an improved electromagnetic field by almost nine times^[^ ^88^ ^]^ (Figure 4c). The LSPR impact was enhanced by the core–shell structure with SiO_2_ covering, indicating that more sophisticated nanoplatforms might promote greater therapeutic benefits. However, the introduction of toxic irons causes potential biosafety issues.^[^ ^83a^ ^]^ Thus, methods such as “transforming trash into treasure” urgently needed to overcome this problem.

### 3.4 Heterojunctions Construction of Ti‐Based Nanosonosensitizers

A prospective method to increase the effectiveness of sonosensitizers is the development of heterojunction, which can be classified into semiconductor–semiconductor (S–S) heterojunctions and semiconductor–metal (S–M) heterojunctions based on the conductivity of material. S–S heterojunctions comprise type I/II/III, P‐N, and Z‐scheme junctions in terms of the electron transfer mechanism. Numerous intrinsic p‐ or n‐type semiconductors with varying mechanical, physical, and chemical properties can be created using surface engineering techniques including doping and defect engineering, offering an abundance of raw materials for the creation of S–S heterojunctions. The positions of the electronic band edges and the work functions of two semiconductors are often crucial parameters to ascertain the electron transfer mechanism of a semiconductor heterojunction, which directly impacts its catalytic activity.^[^ ^96^ ^]^ Furthermore, S–M heterojunctions include Schottky, Ohmic, and LSPR‐mediated junctions. The characteristics of semiconductor‐metal contacts, including Schottky junction and Ohmic contact, are linked to the type of the semiconductor and the relative work function between the metal and semiconductor.^[^ ^96^ ^]^

**Figure 5:**
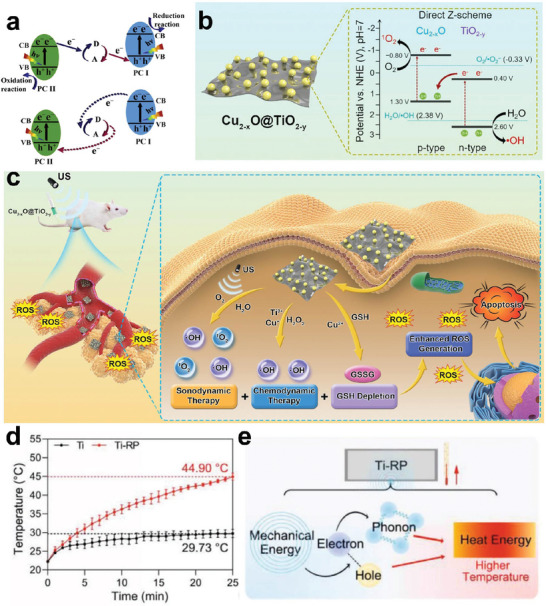
Heterojunction in Ti‐based sonosensitizer. a) Schematic illustration of charge transfer (up) and unwanted charge‐transfer mode (down) in Z‐scheme heterojunction photocatalysts. Reproduced with permission.^[^ ^98^ ^]^ Copyright 2020, Cell Press. b,c) Fabrication and function of Cu_2−x_O@TiO_2−y_ Z‐scheme junctions. Reproduced with permission.^[^ ^100^ ^]^ Copyright 2022, Elsevier. d) Thermal characteristics of Ti‐RP under US activation. e) Mechanism of the sonothermal effect. Reproduced with permission.^[^ ^101^ ^]^ Copyright 2021, Wiley.

By effectively inhibiting the recombination of e^−^–h^+^ and forming multiple reactive active sites, heterojunction nanomedicine could expand substrate selectivity, regulate tumor inhibition microenvironment, and simultaneously maintain the function of single component. On this ground, direct Z‐scheme heterojunction without an electron mediator (g‐C_3_N_4_/TiO_2_ Z‐scheme heterojunction) was firstly reported by Yu et al. in 2013.^[^ ^97^ ^]^ The electrons of semiconductor with higher potential will be transferred to the lower one after contact with each other according to a fundamental design concept. The consequent band bending of two semiconductors provides the foundation for the combination of electron–hole pairs^[^ ^98^ ^]^ (**Figure** **5**a). Semiconductors can be classified as n‐ or p‐type semiconductors depending on the nature of the main carrier. The predominant carriers in n‐ and p‐type semiconductors are electrons and holes, respectively. For example, Shen et al. recently designed N‐CD@TiO_2−x_ p‐n junctions as high‐efficacy sonosensitizers.^[^ ^99^ ^]^ Pyridine N‐doped carbon dots and oxygen‐deficient TiO_2−x_ nanosheets serve as the p‐type and n‐type semiconductor, respectively. Compared with TiO_2_, the ROS generation of p‐n junctions platform increased by about 4.5‐fold under US excitation via the carrier migration mechanism of Z‐scheme. Correspondingly, Shen's group developed another novel non‐stoichiometric Cu_2−x_O@TiO_2−y_ Z‐type heterojunctions for SDT‐CDT synergistic therapy.^[^ ^100^ ^]^ Such heterojunctions could provide plenty of functionalities for improving therapeutic outcomes, which attribute to the more negative CB potential of Cu_2−x_O (for ^1^O_2_ generation), more positive VB potential of TiO_2−y_ (for •OH generation) (Figure 5b,c). Furthermore, Cu_2−x_O@TiO_2−y_ under US irradiation has completely eliminated local tumors and limited tumor pulmonary metastasis due to the enhanced carrier separation dynamics and Fenton function catalyzed by Cu^+^ and Ti^3+^ ions. These state‐of‐the‐art works substantiate the unprecedented potential of Z‐type heterojunctions as next‐generation sonosensitizers.

Moreover, the titania‐coated Au nanoplate heterostructures designed by Yang et al.^[^ ^92^ ^]^ and Ti‐metal‐red phosphorus (Ti‐RP) engineered by Wu et al.^[^ ^101^ ^]^ manifested feasible strategies for using S–M heterojunction (Figure 5d). Noble metals in heterojunctions also serve as excellent electronic mediators due to their good electrical conductivity. In other words, metals served as mediators to move electrons from one CB to another in addition to separating the e–h^+^ couples through LSPR, increasing the redox potentials for the generation of ROS. Moreover, the Ti‐RP integrated the sonothermal effect with SDT by increasing temperature from 22.30 to 44.90 °C under the continuous US (20 °C higher than Ti metal) (Figure 5e).

Represented by Ti_2_C_3_, some layered nanomaterials featured excellent superior conductivity would provide a broad range of material candidates for the engineering of S−M heterojunctions. The rigorous requirements for metals and semiconductors have greatly hindered the development of S−M heterojunctions in the biomedical sector, despite their enormous potential. In summary, heterojunction nanomedicine has distinctive biological features and promotes the creation of sophisticated nanoplatforms for the cancer treatment.

Recent research concluded the van der Waals heterojunction in PDT application.^[^ ^102^ ^]^ When various nanomaterials are integrated into a single nanoplatform, heterojunctions are created that have capabilities for catalysis, detection, nanobiosensing, and multimodal imaging.^[^ ^103^ ^]^ The separation of electrons and holes in heterojunctions following stimulation and their transport to the conduction band and valence band result in increased redox potentials, which in turn cause the production of ROS.^[^ ^104^ ^]^ The band structure of the sonosensitizer can be further modified by logically creating the heterojunction. Heterojunction nanomedicines also feature several reactive sites that can increase substrate selectivity and result in compelling and secure disease therapy.

### 3.5 TME Modulation for Enhanced SDT

One of the key elements influencing the formation of ROS in SDT is the hypoxic tumor microenvironment.^[^ ^105^ ^]^ SDT is an oxygen consumption mechanism that increases the hypoxic environment while also occasionally struggling for oxygen supplies.^[^ ^90^, ^106^ ^]^ There are three main approaches to solving this problem: I) delivering oxygen to TME;^[^ ^107^ ^]^ II) generating oxygen in TME;^[^ ^108^ ^]^ and III) targeting tumor hypoxia.^[^ ^109^ ^]^ In a word, the investigation into remolding the TME has reached a pinnacle since it has the potential to indirectly improve the effectiveness of cancer therapy.

Generally, the high heat stimulated by near‐infrared (NIR) I/II laser in PTT would accelerate the blood flow, thus enhancing tumor oxygenation. Utilizing the mild photothermal effect in SDT is an effective approach to alleviate hypoxia environment. For instance, coordinating PTT with SDT, Cheng and his colleagues created TiN nanodots that responded to both NIR‐II and US radiation.^[^ ^30^ ^]^ TiH_1.924_ nanodots created by Liu et al. were also functioned in the light of this mode.^[^ ^31^ ^]^

**Figure 6:**
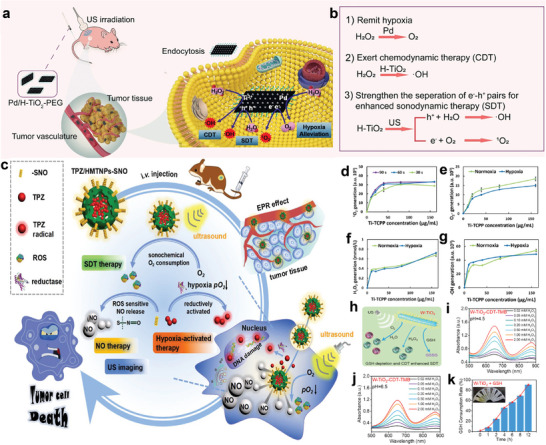
Modulation of tumor microenvironment. a) In vivo hypoxia amelioration of Pd/H‐TiO_2_‐PEG. Reproduced with permission.^[^ ^111^ ^]^ Copyright 2022, Springer Nature. b) The multifunction characteristics of Pd/H‐TiO_2_‐PEG in sonodynamic treatment. c) Schematic illustration of all‐in‐one theranostic platform. Reproduced with permission.^[^ ^35b^ ^]^ Copyright 2018, Elsevier. d–g) Concentration‐dependent ^1^O_2_, O^2−^, H_2_O_2_, and •OH generation after US excitation under normoxic and hypoxic conditions. Reproduced with permission.^[^ ^113^ ^]^ Copyright 2021, Springer Nature. h) Enhanced ROS generation by W‐TiO_2_ nanorods. i,j) The chemodynamic performances of W‐TiO_2_ nanorods utilizing the TMB probe at pH 4.5 or 6.5. k) GSH consumption test through the 5'‐dithiobis‐(2‐nitrobenzoic acid) solution assays. Reproduced with permission.^[^ ^114^ ^]^ Copyright 2021, American Chemical Society.

Generating oxygen in TME is also a hot spot strategy. For example, a defected 2D Pd/H‐TiO_2_ NSs designed by our group possessed catalase‐like activity, a common O_2_‐generating technique,^[^ ^80^, ^110^ ^]^ alleviating the hypoxic condition and enhancing SDT and CDT efficacies simultaneously^[^ ^111^ ^]^ (**Figure** **6**a,b). Similarly, Zhou et al. synthesized TiO_2_‐Fe_3_O_4_@PEG Janus nanostructure, in which Fe_3_O_4_ serves as nanoenzyme and endows nanosonosensitizers with Fenton‐catalytic activity, furthermore narrowing the band gap of TiO_2_.^[^ ^112^ ^]^

Modulating the TME may not guarantee the initiation of other pathways for the survival of cancer cells due to the complexity and diversity of tumor formation. Thus, an alternative way for addressing the detrimental effects of hypoxia is to load cytotoxic chemicals that become lethal when exposed to a particular type of hypoxia.^[^ ^35^, ^115^ ^]^ For example, Zhang et al. utilized tirapazamine (TPZ) as a hypoxic cytotoxin and hollow mesoporous titanium dioxide nanoparticles (HMTNPs) as a carrier for the fabrication of TPZ/HMTNPs‐SNO therapeutic agents modifying with S‐nitrosothiol (R‐SNO)^[^ ^35b^ ^]^ (Figure 6c). The highly reactive TPZ radical could trigger radical‐mediated deoxyribonucleic acid breakage and destroy hypoxic cells in a hypoxic microenvironment. Lu et al. chose the hypoxia‐activated prodrug AQ4N as a cytotoxic chemical in a mesoporous titanium dioxide delivery system in another paradigm.^[^ ^116^ ^]^ Under US stimulation, the anoxic environment transforms AQ4N into harmful toxins, sparking the anti‐tumor effectiveness of SDT and reducing the negative circulation of hypoxia.

Not merely occurring in photodynamic therapy, researches show type I/II reactions also happen in SDT. Specifically, type I SDT would undergo an O_2_‐independent modality when oxygen is running out.^[^ ^117^ ^]^ For example, a tablet‐like TiO_2_/C MOF with hypoxia‐tolerant qualities was established by Zhang et al. to produce ROS continuously under repeated US irradiation.^[^ ^110b^ ^]^ Besides, Huang et al. designed a Ti‐based MOF platform, nucleus‐targeted Ti‐tetrakis (4‐carboxyphenyl) porphyrin, to produce significant amounts of ROS in an oxygen‐independent mode^[^ ^113^ ^]^ (Figure 6d–g). Type I SDT offers a fresh paradigm for overcoming oxygen‐insufficient conditions, which typically function in severely hypoxic malignancies like orthotopic pancreatic cancer.

Notably, the accumulation of L‐Glutathione (GSH) in TME would also restrain the ROS production,^[^ ^118^ ^]^ severely impairing the therapeutic effectiveness of SDT. On this ground, reducing GSH levels in the tumor region becomes another promising method. For example, W‐doped TiO_2_ nanorods featuring GSH‐depleting activities could overcome the SDT resistance in response to endogenous GSH^[^ ^114^ ^]^ (Figure 6h–k, **Table** **1**). Due to the insufficient oxygen content in TME, O_2_‐dependent therapies, including SDT, show limited performance in cancer treatment. Therefore, increasing the oxygen content in tumor site or decrease the oxygen consumption may be the effective means for the improvement of therapeutic effect.

**Table 1:** Different representative optimization strategies of Ti‐based sonosensitizers

Materials	Sonosensitizer	Optimization method	Ref.
Fe‐TiO_2_	TiO_2_	LSPR	[61b]
Au‐TiO_2_	TiO_2_	LSPR	[91]
Au‐TiO_2_	TiO_2_	LSPR	[67a]
Au@TiO_2_	TiO_2_	LSPR	[92]
PT‐TiO_2_(DOX)	TiO_2_	LSPR	[80]
CD@Ti_3_C_2_T_x_	Ti_3_C_2_	LSPR	[93]
HABT‐C@HA	TiO_2_	LSPR	[89]
TiO_2_@TiO_2−x_	TiO_2_	Oxygen defects	[61a]
TiO_1−x_	TiO_1−x_	Oxygen defects	[57]
Ti_3_C_2_@TiO_2−x_	Ti_3_C_2_	Oxygen defects	[24]
Ti‐S‐TiO_2−x_	TiO_2−x_	Oxygen defects	[28]
HABT‐C@HA	TiO_2_	Oxygen defects	[89]
D‐MOF(Ti)	MOF(Ti)	Oxygen defects	[119]
Pt‐TiO_2_(DOX)	TiO_2_	Oxygen defects	[80]
PPBP‐B‐TiO_2_	TiO_2_	Oxygen defects	[81]
H–Ti_3_C_2_‐PEG	Ti_3_C_2_	Oxygen defects	[26]
Pd/H‐TiO_2_	TiO_2_	Oxygen defects	[111]
NH_2_‐MIL	Ti‐MOF	Oxygen defects	[120]
TiO_2_‐GR	TiO_2_	Heterojunction	[62]
Fe_3_O_4_@TiO_2_	TiO_2_	Heterojunction	[121]
Ti‐RP	Ti	Heterojunction	[101]
Cu_2−x_O@TiO_2−y_	TiO_2−y_	Heterojunction	[100]
Au@TiO_2_	TiO_2_	Heterojunction	[92]
N‐CD@TiO_2−x_	TiO_2−x_	Heterojunction	[99]
Pt‐TiO_2_(DOX)	TiO_2_	Heterojunction	[80]
NH_2_‐MIL	Ti‐MOF	Heterojunction	[120]
Ti‐TCPP	Ti	Modulation of TME	[113]
TiO_2_/C	TiO_2_	Modulation of TME	[110b]
Pd/H‐TiO_2_	TiO_2_	Modulation of TME	[111]
PVP‐TiN	TiN	Modulation of TME	[30]
TPZ/HMTNPs‐SNO	TiO_2_	Modulation of TME	[35b]
HABT‐C@HA	TiO_2_	Modulation of TME	[89]
TiO_2_‐Fe_3_O_4_@PEG	TiO_2_	Modulation of TME	[112]
W‐TiO_2_	TiO_2_	Modulation of TME	[114]
Nb_2_C/TiO_2_/BSO‐PVP	TiO_2_	Modulation of TME	[122]
Pt–TiO_2_	TiO_2_	Modulation of TME	[80]
C‐TiO_2_/TPZ@CM	TiO_2_	Modulation of TME	[49]
C‐TiO_2_/AIPH@PM	TiO_2_	Modulation of TME	[123]

### 3.6 Ti‐Based Nanosonosensitizers for Synergistic Cancer Therapy

SDT combined with other strategies can significantly improve theranostic outcomes and overcome the limitations of monotherapy. In this part, we aim to concentrate on the latest reports and especially highlight the integration between novel techniques such as oxygen‐deficient engineering, plasmon resonance modulation, and other strategies, thus presenting the future directions to advance the efficiency of tumor elimination.

#### 3.6.1 Ti‐Based Nanosonosensitizers for SDT‐Chemo Synergistic Therapy

Since the design of specific mesoporous TiO_2_ structure, the nanoplatforms can act as a drug carrier while generating ROS under US activation. Based on that, different modifying or coating approaches endow nanosensitizers with decreased toxicity and controlled release performance, efficiently eliminating tumor. Optimized strategies for improving SDT efficiency (oxygen‐deficient technique or combining TiO_2_ with noble metals) are similarly competent for the synergistic effects of high‐performance dual‐modal therapy. For example, Lin et al. firstly reported a hydrogenated hollow Pt‐TiO_2_ composite system, which simultaneously served as SDT agent and a reservoir to load doxorubicin.^[^ ^80^ ^]^ An oxygen‐deficient layer formed on the TiO_2_ surface as a result of the hydrogenation reduction of Pt‐TiO_2_. On the one hand, Pt NPs function as a catalase‐like enzyme to lessen resistant of chemotherapy. On the other hand, it generated enough oxygen in a low‐oxygen setting to support the formation of ROS brought on by SDT.

N‐doped TiO_2_/C‐PEG were successfully synthesized via calcining MIL‐125 (Ti) and MIL‐125 (Ti)‐NH_2_ followed by coating with PEG,^[^ ^124^ ^]^ which exhibited outstanding chemotherapy and sonodynamic effect due to the MOF‐derived metal/C hybrid structures and TiO_2_ bandgap reduction (from 3.2 to 2.4 eV) by N‐doping. Furthermore, the TPZ was effectively carried by the abovementioned platform and killed cells together with ROS.

The ongoing focus of oncology research is overcoming drug resistance and increasing the susceptibility of tumor cells to chemotherapy. US can boost the specific absorption of chemotherapy medications into cancer cells, minimizing the toxicity and adverse effects on healthy cells and tissues. Therefore, it was concluded that the SDT‐chemotherapy technique has the potential to encourage further clinical translation in the field of cancer treatment.

#### 3.6.2 Ti‐Based Nanosonosensitizers for SDT‐CDT Synergistic Therapy

In recent years, CDT has gained popularity as a cancer treatment due to the Fenton/Fenton‐like reactions that turn high levels of hydrogen peroxide (H_2_O_2_) into ROS.^[^ ^125^ ^]^ Despite the huge potential of CDT, the inherent limitations such as the high expression of GSH and micro‐acidity of the tumor sites impede the effectiveness of single CDT. On the other hand, TiO_2_ NPs also suffer from low ROS productivity owing to fast e^−^–h^+^ recombination. Therefore, combining CDT‐SDT may be able to address the issues to produce desired theranostic results.

**Figure 7:**
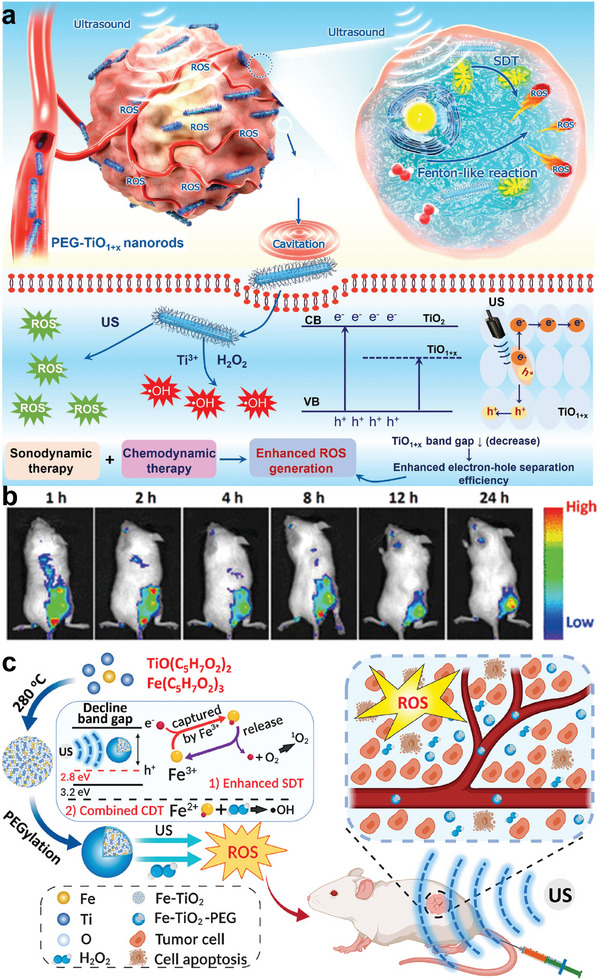
Ti‐based nanosonosensitizers for synergistic SDT‐CDT. a) Schematic diagram of TiO_1+x_ nanorods for SDT/CDT. Reproduced with permission.^[^ ^57^ ^]^ Copyright 2020, American Chemical Society. b) The fluorescence images of 4T1 tumor‐bearing mice after an intravenous injection of Cy5.5‐labeled Fe‐TiO_2_ nanodots. c) Schematic illustration of enhanced sonodynamic and chemodynamic outcomes for dual‐modal imaging‐guided SDT/CDT combination treatment using Fe‐TiO_2_ nanodots. Reproduced with permission.^[^ ^61b^ ^]^ Copyright 2020, American Chemical Society.

For example, Ding et al. designed engineering defected Pd/H‐TiO_2_ nanosheets via one‐pot Pd‐catalyzed hydrogenation strategy.^[^ ^111^ ^]^ Tiny Pd ingredient and an oxygen‐deficient structure provide nanosheets with catalase‐like activity and Fenton‐like functionality, resulting in a nanoplatform with triple antineoplastic properties that improves single SDT efficacies. Cheng et al. developed titanium monoxide nanorods (TiO_1+x_ NRs) with an ultrafine rod‐like shape that displayed horseradish‐peroxidase‐like nanozyme activity, which can produce •OH from H_2_O_2_ in the tumor site to achieve CDT^[^ ^57^ ^]^ (**Figure** **7**a). Cheng's group, in another study, designed iron‐doped titanium oxide nanodots (Fe‐TiO_2_ NDs) for the collaboration between Fenton‐catalytic function and SDT^[^ ^61b^ ^]^ (Figure 7b,c).

CDT struggles to have a significant anti‐cancer effect due to the absence of sufficient H_2_O_2_ concentration. Herein, Jing et al. synthesized 2‐dimentional Ti_3_C_2_/CuO_2_@BSA nanosheets that achieve in situ Cu^2+^‐mediated H_2_O_2_ generation due to the pH‐triggered decomposition of CuO_2_, ultimately oxidizing Ti_3_C_2_ to produce TiO_2_ nanosonosensitizers.^[^ ^126^ ^]^ It also enhanced the separation of electrons and holes beneath the carbon matrix upon oxidation, producing various therapeutic benefits. After US irradiation, the Fenton‐like reaction was intensified, simultaneously coordinating CDT/SDT effect both in vitro and in vivo.

#### 3.6.3 Ti‐Based Nanosonosensitizers for SDT‐PTT Synergistic Therapy

Photothermal therapy (PTT), one of the most promising cancer treatments, kills tumor cells with photothermal transformation performance and exhibits high selectivity and non‐invasive properties.^[^ ^127^ ^]^ However, PTT still has its inherent limitation. For instance, NIR laser will undoubtedly scatter and be absorbed during the therapy process, it is powerless to remove the deep‐seated tumor.^[^ ^128^ ^]^

**Figure 8:**
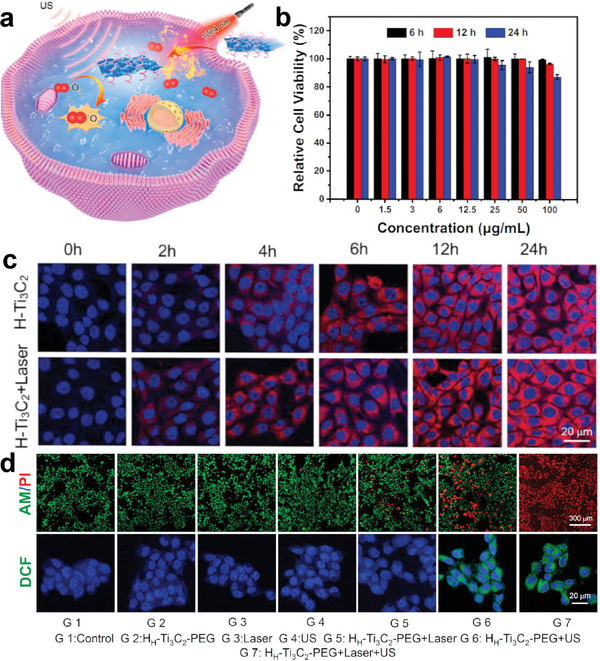
Ti‐based nanosensitizers for SDT‐PTT. a) Schematic diagram of H_H_‐Ti_3_C_2_‐PEG NSs for the PTT‐SDT. b) Relative cell viability of 4T1 cells treated with different doses of H_H_‐Ti_3_C_2_‐PEG NSs for 6, 12, and 24 h. c) Confocal pictures of 4T1 cells cultured with Cy5.5‐conjugated H_H_‐Ti_3_C_2_‐PEG NSs for varying periods of time with or without 1064 nm laser irradiation. d) Calcein‐AM/propidium iodide assays and intracellular ROS levels treated with different agents (G1–G7). Reproduced with permission.^[^ ^26^ ^]^ Copyright 2022, Elsevier.

Considering the oxygen consumption of SDT, O_2_ supply is required. PTT accelerate blood flow by heating the local tumor environment, providing a feasible strategy to enhance overall ROS productivity.^[^ ^29^ ^]^ For example, Cheng et al. designed a deficient Ti_3_C_2_ MXene NSs through chemical exfoliation and high‐temperature methods. The mild photothermal effect can not only promoted the endocytosis of as‐synthesized nanosheets, but also increased blood supply and relieved hypoxia in the TM^[^ ^26^ ^]^ (**Figure** **8**a–d). The defect structure could significantly facilitate the separation of electrons and holes from the energy‐band structure of Ti_3_C_2_ NSs under US irradiation. Hu et al. integrated P‐DOX and P‐Aspirin into the as‐prepared mTiO_2_‐PPy NCs for SDT‐PTT synergistic therapy.^[^ ^55^ ^]^ In response to the acidic tumor microenvironment, this exquisite nanoplatforms could accomplish NIR‐US dual‐response modality and accelerate prodrug maturation behavior. Compared to SDT and PTT alone, SDT‐PTT greatly improved the treatment effectiveness of malignancies, allowing for total tumor eradication without recurrence.

#### 3.6.4 Ti‐Based Nanosonosensitizers for SDT‐Immunosynergistic Therapy

Cancer immunotherapy is another exciting and rapidly expanding field in cancer treatment, which focuses particularly on enhancing the stimulation of an anticancer immune response while minimizing the immunosuppressive features of the TME.^[^ ^129^ ^]^ Tumor immunotherapy that works offers a more specialized and targeted approach to cancer treatment. For instance, immune checkpoint blockade therapy is one of the most encouraging strategies by influencing the CTLA‐4, PD‐1/PD‐L1 axis and disrupting co‐inhibitory T‐cell signaling.^[^ ^130^ ^]^ However, immune therapy approaches still face several safety and process‐related issues, particularly when it comes to dosage ambiguity. As such, these restrictions have prompted a few current initiatives to develop therapeutic techniques to improve responsiveness to treatment and get around resistance and toxicity.^[^ ^131^ ^]^ Based on these inherent limitations, immunotherapy undergoes a synergistic strategy, such as in combination with SDT, to reach more diversified functions.

**Figure 9:**
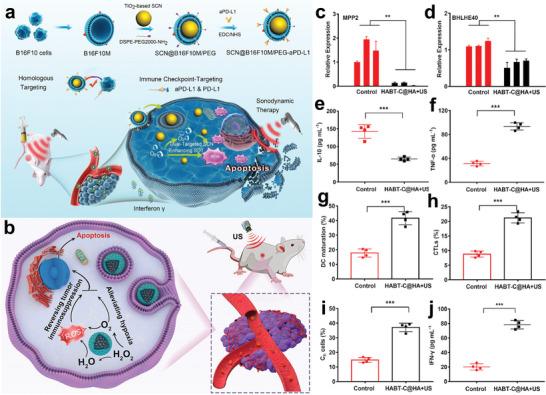
Ti‐based nanosensitizers for SDT‐immunosynergistic therapy. a) Schematic illustration of synthesis and immune checkpoint targeted enhanced SDT of SCN@B16F10M/PEG‐aPD‐L1a. Reproduced with permission.^[^ ^36^ ^]^ Copyright 2021, American Chemical Society. b) Schematic diagram of synthesis and immunosuppression‐SDT therapy of HABT. c,d) The expression of MPP2 and BHLHE40 genes by qPCR testing. e,f) Cytokine (IL‐10 and TNF‐a) concentrations in serum. g) Proportions of maturing DCs (CD80^high^CD86^high^) from the flow cytometry measurements (gating on CD11c^+^). h,i) Th cells and CTLs infiltrations in tumors 3 days after therapy. j) Serum IFN‐g levels treated with different therapies. Reproduced with permission.^[^ ^89^ ^]^ Copyright 2021, American Chemical Society.

For example, Qiu et al. designed a homology and immune checkpoint dual‐targeted SCN@B16F10M/PEG‐aPD‐L1a nanoplatform via loading programmed cell death ligand 1 antibody (aPD‐L1) and malignant melanoma cell membrane on the TiO_2_‐based sonosensitizers^[^ ^36^ ^]^ (**Figure** **9**a). The sonosensitizer was not recognized as foreign and was quickly removed by the reticuloendothelial system due to the encapsulation of functional cell membranes. Furthermore, the aPD‐L1 as an immune checkpoint after interacting with PD‐L1 is expressed on the tumor cells, ultimately enhancing the CD8+ T‐cell effector activity.^[^ ^132^ ^]^ The above nanoplatform exhibited precise targeting effects by virtue of the combination of two strategies and realized high tumor cell uptake simultaneously. Effective cancer therapy faces significant obstacles in the simultaneous relief of hypoxia and immunosuppression in the TME. Based on that, Liu et al. modified hollow black TiO_2_ nanosphere (HABT) with Au nanoparticles and carbon dots, which possessed triple‐enzyme mimetic activity and alleviated hypoxia in TME by producing sufficient oxygen and generating abundant ROS under US stimulation.^[^ ^89^ ^]^ It should be noted that O_2_ is an essential source of ROS generation in the SDT process. Therefore, the modulation of O_2_ supply enhanced the efficacy of SDT. What's more, the as‐synthesized carbon dots‐HABT can effectively inhibit immunosuppressive mediator expression and reverse the immunosuppression in the TME, providing an SDT/immunotherapy synergistic paradigm (Figure 9b–j).

#### 3.6.5 Ti‐Based Nanosonosensitizers for Other Representative Synergistic Therapy

##### 3.6.5.1 Autophagy

**Figure 10:**
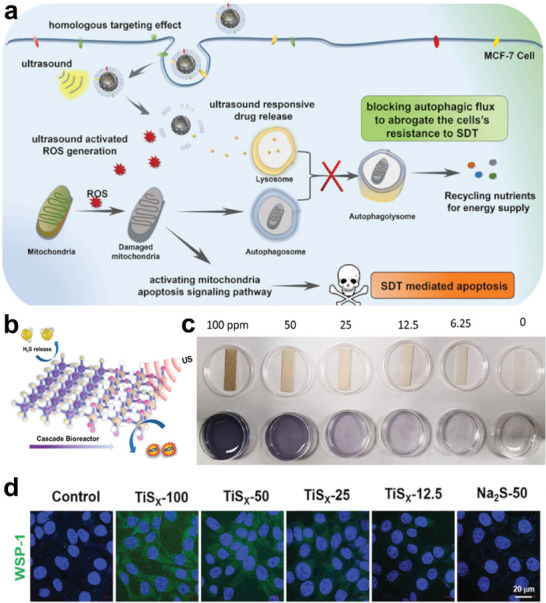
Ti‐based nanosonosensitizers for autophagy and gas therapy. a) Schematic illustration of CCM‐HMTNPs/HCQ nanoplatform for enhanced SDT via autophagy regulation strategy. Reproduced with permission.^[^ ^70^ ^]^ Copyright 2019, American Chemical Society. b) Schematic diagram of H_2_S release and sonodynamic procedure of PEG‐TiS_X_ NSs. c) H_2_S release property of PEG‐TiS_X_ NSs with different concentrations. d) Performance of H_2_S release in vitro with PEG‐TiS_X_ NSs with different concentrations by the WSP‐1 probe. Reproduced with permission.^[^ ^135^ ^]^ Copyright 2022, Wiley.

In general, the process of cytoplasmic elements degradation within lysosomes is known as “autophagy.”^[^ ^133^ ^]^ Studies have demonstrated that autophagy has a variety of roles, such as anti‐ageing, cell death, tumor suppression, and so on. The contribution of autophagy turns complicated in some circumstance.^[^ ^134^ ^]^ Researchers describe it as a double‐edged sword, for autophagy could cooperate, aggravate, or antagonize apoptosis in the field of cancer therapy. Interestingly, SDT could function as an autophagy‐inducer to a large extent. Zhang et al. reported hollow mesoporous TiO_2_ nanoparticles coated with cancer cell membrane and loaded on autophagy inhibitor (hydroxychloroquine sulfate, HCQ).^[^ ^70^ ^]^ The biomimetic surface functionalization endows sonosensitizer with the homologous targeting ability. By actively identifying and focusing on the tumor site, it can evade phagocytosis by macrophages. In an effort to lessen the cell resistance to SDT, the release of HCQ would inhibit autophagic flow and restrict the provision of nutrients from the wounded organelles under US stimulation. Moreover, HCQ could normalize tumor vessels, alleviate hypoxia in the TME and ultimately enhance SDT for cancer therapy (**Figure** **10**a). This research gives a view of a new therapeutic paradigm for targeting autophagy in SDT.

##### 3.6.5.2 Gas Therapy

By using gaseous chemicals including NO, SO_2_, CO, CO_2_, and H_2_S, gas therapy exerts anti‐cancer effect via interfering with cellular function or inducing inflammation, thus named “green” therapy.^[^ ^136^ ^]^ For example, Cheng et al. prepared titanium sulfide nanosheets (TiS_X_ NSs) for sequential gas‐sonodynamic cancer therapy.^[^ ^135^ ^]^ As H_2_S donors, TiS_X_ NSs can burst and emit H_2_S gas. Following the formation of H_2_S, TiS_X_ NSs rapidly deteriorate to become S‐defective and partially oxidize into TiO_X_ on their surface, giving them advantageous sonodynamic characteristics when exposed to US radiation (Figure 10b–d). Moreover, NO plays a virtue role due to the remarkable therapeutic effect. High concentration NO (>1 µm) could efficiently kill tumor cells and cooperatively augment other therapies such as PTT, PDT, chemotherapy, etc.^[^ ^137^ ^]^ Although NO shows outstanding performance in a series of pathophysiological activities, its short half‐life in buffer or plasma remains a significant challenge.^[^ ^138^ ^]^ It urgently needs an appropriate carrier to achieve precise delivery as well as controlled release. Lin et al. designed a multifunctional black mesoporous TiO_2_ (BMT) nanovaccine for gas therapy, SDT and immunotherapy.^[^ ^38a^ ^]^ L‐arginine (LA) was loaded on BMT which served as the exogenous NO supplementation, and BTM as acoustic sensitizer for sonodynamic treatment. After the exogenous stimulus of US, BMT, and LA could be triggered simultaneously and produce singlet oxygen and NO gas, respectively. Most importantly, the introduction of immune checkpoint inhibitor PD‐L1 antibody (*α*PD‐L1) induced immune response, killed primary tumors and inhibited metastatic tumors.

#### 3.6.6 Ti‐Based Nanosonosensitizers for Piezoelectric Therapy

Piezoelectric materials, such as ZnO, MoS_2_, and BaTiO_3_, have been widely studied and discussed because of the charge‐carrier characteristics.^[^ ^139^ ^]^ The piezoelectric effect of these semiconductors is a coupling of mechanics and electric polarization, originating from the non‐centrosymmetric structure. Piezoelectric materials produce electricity when strained^[^ ^140^ ^]^ and can be utilized to establish a dynamically renewed built‐in electric field under mechanical force. The carrier separates and energy band bends under the mechanical force of US, leading to an enhancement of ROS generation activity.

**Figure 11:**
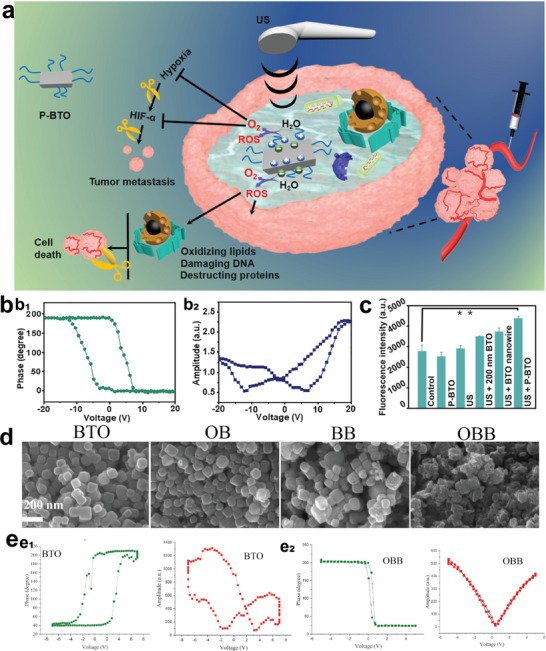
Ti‐based nanosonosensitizers for piezoelectric therapy. a) Schematic of the piezocatalytic therapy. b‐b_1_): Phase curve of P‐BTO observed from piezo‐response force microscopy (PFM); b_2_) Amplitude curve of P‐BTO. Reproduced with permission.^[^ ^142^ ^]^ Copyright 2021, American Chemical Society. c) ROS production ability of different groups. d) SEM images of BTO, OB, BB, and OBB NPs. e‐e_1_) Phase curve of BTO and OBB obtained from PFM; e_2_) Amplitude curve of BTO and OBB. Reproduced with permission.^[^ ^40^ ^]^ Copyright 2022, Elsevier.

For instance, ultrasmall tetragonal BaTiO_3_ would enter an imbalanced charge state on the surface under US irradiation. The excess charges can induce redox reaction behaviors including reacting with the water molecules to generate oxygen, interacting with water molecules or oxygen to generate ROS.^[^ ^141^ ^]^ At present, the application of BaTiO_3_ in cancer treatment is still in its fancy. Liang and his co‐workers designed an ultrasmall tetragonal DSPE‐PEG_2000_ coated BaTiO_3_ (P‐BTO) nanoparticles. Surface charge on the P‐BTO under US vibration may trigger a series of redox reactions that eventually produce O_2_ and ROS, reversing the hypoxia of the TME and effectively killing tumor cells^[^ ^142^ ^]^ (**Figure** **11**a–c). Our group provided a novel paradigm of dynamic therapy between piezocatalysis and cancer treatment.^[^ ^44^ ^]^ We designed an injectable thermosensitive hydrogel (thermogel) and realize tumor eradication under US irradiation. The thermosensitive hydrogel would significantly promote US‐triggered cytotoxicity and piezocatalytic tumor elimination, coupled by good therapeutic biosafety according to in vitro and in vivo assessment. This modality differs from traditional sonoluminescence‐activated sonodynamic therapy in a number of important ways, including a more stable sonosensitizer and dynamical control of redox processes.

It has been reported that incorporating noble metals into BTO would trap the excited electrons and enhance the separation of e^−^/h^+^ pairs.^[^ ^143^ ^]^ As such, Wang et al. efficiently suppressed the growth of ovarian cancer by doping Bi on the oxygen‐deficient BaTiO_3_^[^ ^40^ ^]^ (Figure 11d,e). It can be speculated that the piezocatalytic treatment strategy for orthotopic tumor as well as patient‐originated tumor xenograft will be the focus of future research since it is highly significant for the possible clinical use (**Table** **2**).

**Table 2:** Summary of SDT‐based synergistic therapy

Sonosensitizers	Synergistic therapy	Ref.
Pt‐TiO_2_	SDT‐chemotherapy	[80]
MTN@DTX‐CD		[35a]
pPBA@TNP‐DOX		[144]
TiO_2_:Gd@DOX/FA		[64]
Fe_3_O_4_@TiO_2_‐DOX		[121]
Fe_3_O_4_‐NaYF_4_@TiO_2_		[63]
RBC‐mTNPs@AQ_4_N		[142]
TKD@RMPB		[145]
N‐TiO_2_/C‐PEG@TPZ		[124]
Pd/H‐TiO_2_		[111]
C‐TiO_2_/TPZ@CM		[49]
CD@Ti_3_C_2_Tx HJs	SDT‐PTT	[93]
Ti_3_C_2_@TiO_2−x_		[92]
Au NPL@TiO_2−x_		[146]
TiO_2_@TiO_2−x_		[61a]
PPBP‐B‐TiO_2_		[81]
H–Ti_3_C_2_‐PEG		[26]
Ti‐S‐TiO_2−x_		[28]
MnO_X_/TiO_2_‐GR		[62]
Ir‐B‐TiO_2_@CCM		[147]
TiH_1.924_		[31]
TiN		[30]
DOX@mTiO_2_‐PPy		[55]
PEG‐TiO_1+x_	SDT‐CDT	[57]
Fe‐TiO_2_		[61b]
V‐TiO_2_		[61c]
D‐MOF(Ti)		[119]
Ti_3_C_2_/CuO_2_@BSA		[126]
TiO_2_‐Fe_3_O_4_@PEG		[112]
SCN@B16F10M/PEG‐aPD‐L1	SDT‐Immunotherapy	[36]
HABT‐C@HA		[89]
TiO_2_‐Ce6‐CpG		[148]
Cu_2−x_O‐BaTiO_3_	Piezotronic‐enhance SDT	[42]
P‐BTO		[142]
OBB		[40]
Au@BTO		[149]
T‐BTO		[44]
CCM‐HMTNPs/HCQ	SDT‐autophagy	[70]
P‐BTO	SDT‐gas therapy	[142]
TiSX		[135]
mTiO_2_@PPY‐HNK	SDT‐PTT‐Chemotherapy	[65]
TPZ/HMTNPs‐SNO	SDT‐Chemotherapy‐Gas therapy	[69]
BMT@LA NCs	SDT‐Immunotherapy‐Gas therapy	[38a]
MnTiS‐PEG	SDT‐Immunotherapy‐Gas therapy	[150]

## 4 Ti‐Based Nanosonosensitizers for Other Diseases

**Figure 12:**
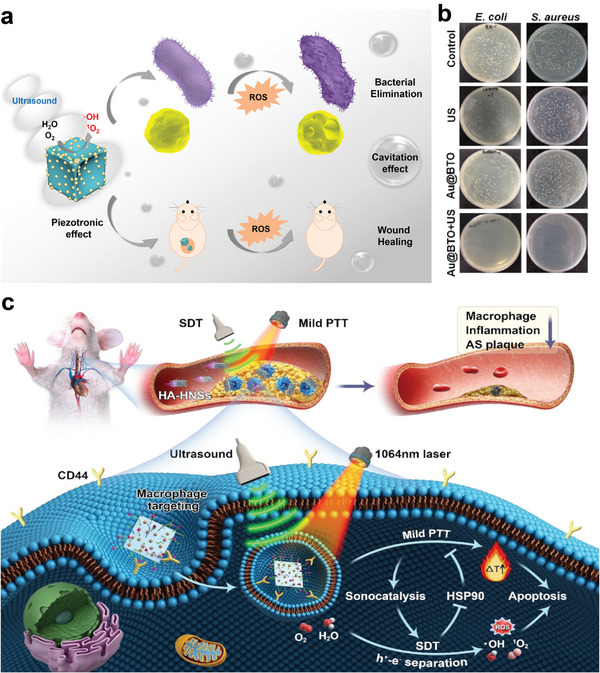
Ti‐based nanosonosensitizers for other diseases beyond tumor. a) Schematic diagram of Au@BTO‐based piezoelectric NCs for sonodynamic antibacterial therapy. Reproduced with permission.^[^ ^149^ ^]^ Copyright 2021, Elsevier. b) The antibacterial performance of Au@BTO against *E. coli* and *S. aureus*. c) Atherosclerotic plaque progression can be prevented by HA‐HNSs‐mediated SDT‐PTT synergistic treatment. Reproduced with permission.^[^ ^152^ ^]^ Copyright 2022, American Chemical Society.

Apart from the application in cancer treatment, Ti‐based nanosonosensitizers are applied in other fields including anti‐bacteria,^[^ ^151^ ^]^ healing wound,^[^ ^149^ ^]^ atherosclerotic plaque,^[^ ^152^ ^]^ etc. For example, Au‐modified piezoelectric BaTiO_3_ nanocubes reported by Li et al. exhibited high antibacterial efficiency against Gram‐negative and Gram‐positive bacteria (**Figure** **12**a,b). A new gel containing of amorphous TiO_x_ nanofibers and Ti_2_C(OH)_2_ nanosheets was created by Yu et al.^[^ ^153^ ^]^ The as‐designed functional gel could capture and trap bacteria easily because the Ti‐OH terminal groups could quickly react with the phosphate groups located in teichoic acid (G^+^ bacteria) and lipopolysaccharides (G^−^ bacteria), thus preventing the invasion of bacteria. Notably, the gel could decompose H_2_O_2_ into O_2_ and alleviate hypoxia and excessive oxidation reaction in the TME, ultimately accelerating angiogenesis and the organizational reshaping procedure. Wu et al. created an oxygen deficiency on Ti‐S‐TiO_2−x_ to combat the recurrence of implant infection, which kills *Staphylococcus aureus* (*S. aureus*) without the use of antibacterial coating, demonstrating an impressive method of SDT‐PTT antibacterial treatment.^[^ ^28^ ^]^

Controlling and regulating cell function is still a difficulty given the complexity of the wound healing process.^[^ ^154^ ^]^ Research shows that US could interact with cells and tissues in a multiplicity of ways and elevate the operation temperature, eventually yielding therapeutic effects.^[^ ^155^ ^]^ US‐mediated therapy has been used to manipulate the recovery of wounds in skin, muscles as well as other tissues.^[^ ^156^ ^]^ On this ground, the pertinent experiments on wound healing utilizing Ti‐based nanosonosensitizers‐involved SDT are thought to be plausible and promising. For example, Wu et al. developed an Au@BaTiO_3_ piezoelectric nanocomposite as a new kind of sonosensitizer for high‐efficient antibacterial efficiency against Gram‐negative and Gram‐positive bacteria. In vitro and in vivo experiments show that the sonodynamic process also encourages fibroblast migration, inducing dermal wound healing.^[^ ^149^ ^]^

Atherosclerosis (AS) as the worldwide chronic arterial disease had a significant mortality rate.^[^ ^157^ ^]^ SDT has been proposed as a potentially feasible strategy in treatment of AS due to the unique ROS generation characteristics.^[^ ^158^ ^]^ SDT directly kills tumor cells by generating cytotoxicity and rupturing cell membrane structures.^[^ ^159^ ^]^ In a recently reported paradigm, Wang et al. designed a CuS/TiO_2_ heterostructure nanosheets (HNSs) with the modification of hyaluronic acid (HA) and PEG (HA‐HNSs) for the prevention of the progression of early AS by removing lesioned macrophages and moderating inflammation.^[^ ^152^ ^]^ Moreover, HA enables HA‐HNSs to target intraplaque proinflammatory macrophages through selectively interacting CD44 (Figure 12c).

Cooperative therapy could balance the therapeutic advantages and compensate the shortcoming.^[^ ^160^ ^]^ In combination with different nanotechniques, such as PTT, PDT, CDT, chemotherapy, immunotherapy, gas therapy, and piezotronics effect, Ti‐based sonosensitizers showed enhanced treating performance both in vivo and in vitro evaluation. Multi‐model and programmable synergistic therapies bring novel paradigms for scientific communities.^[^ ^161^ ^]^

## 5 Biocompatibility and Biosafety of Ti‐Based Nanosonosensitizers for SDT

The therapeutic biosafety and biocompatibility of nanosonosensitizers are pivotal for their continued clinical employment. Ti‐based nanomaterials may have negative consequences on adjacent tissues and organs and potentially induce systemic responses.^[^ ^29^ ^]^ It is necessary to comprehensively evaluate the pharmacokinetic profiles, biocompatibility, bioavailability, and possible long‐term side effects. Consider the case of TiO_2_, Lin et al. decorated hollow Pt‐TiO_2_ nano‐sonosensitizer and assessed its cytotoxicity via standard methyl thiazolyl tetrazolium/3‐[4,5‐dimethylthiazol‐2‐yl]‐2,5‐diphenyltetrazolium bromide test. With a concentration of 500 ppm Ti, cell survival rates in 4T1 cancer cells, HeLa and normal L929 cells were both above 85%, suggesting the negligible toxicity of TiO_2_ in vitro. Furthermore, the healthy mice were intravenously injected with Pt‐TiO_2_ (5, 10, and 20 mg kg^−1^), and the biochemistry assays, H&E staining and metabolism results proved the remarkable biosafety and biocompatibility in vivo.^[^ ^80^ ^]^ Similarly, Ti_3_C_2_ nanosheets,^[^ ^162^ ^]^ TiN NPs,^[^ ^163^ ^]^ TiN, TiH_1.924_ nanodots have been proved to possess superb biosafety. In addition, the optimization of preparation strategy can properly adjust the biocompatibility of materials. For example, defect engineering directly optimizes the sonosensitizers structure without introducing other components, which can guarantee the high biosafety of sonosensitizers. The biocompatibility and biosafety of the inorganic nanosonosensitizers are decreased as a result of changing the TME by incorporating nanosystems that can generate oxygen.^[^ ^90^ ^]^

Ti‐based nanomaterials may still experience long‐term toxicity issues, despite the fact that techniques like surface modification endow nanoplatforms with the targeted ability. With that in mind, ROS‐manipulated drug delivery platform has been developed by Shi et al. for on‐demand release of DTX, which considerably reduced the negative effects of DTX by protecting tumor‐bearing mice from splenic and hematologic damage.^[^ ^35a^ ^]^ Considering the impact of particle size on biosafety, Cheng et al. fabricated ultrasmall Fe‐TiO_2_ nanodots ranging from 2.49 ± 0.74 nm through a thermal decomposition strategy. After PEG modification, Fe‐TiO_2_‐PEG nanodots demonstrate outstanding physiological stability and biocompatibility. What's more, the long‐term biodistribution, blood biochemistry, and H&E staining results showed negligible damage to the main organs due to the tiny size.^[^ ^61b^ ^]^ The toxicity caused by the accumulation of nanomaterials in healthy organs and tissues following systemic distribution has significantly restricted the pre‐clinical transformation of nanoparticles. Future nanotoxicology research must employ a multidisciplinary team approach to achieve an accurate risk assessment (e.g., toxicology, materials science, molecular biology, medicine, and bioinformatics).

## 6 Conclusion and Perspectives

**Figure 13:**
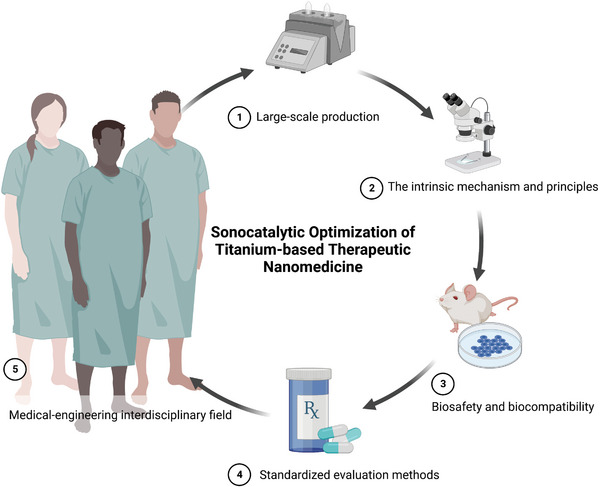
Schematic illustration of the development and challenges of sonocatalytic optimization of titanium‐involved therapeutic nanomedicine, including optimization of synthesis method, research on internal mechanism, systematic biosafety, standardized evaluation methods, improvement of the efficiency of sonosensitizers and promotion of clinical transformation.

Over the past decades, US‐activated therapeutic modality has advanced quickly in recent years as a powerful and non‐invasive anti‐cancer tool, particularly in fundamental research.^[^ ^164^ ^]^ The exploration and optimization of high‐performance Ti‐based inorganic sonosensitizers, including surface modification, defect engineering, plasmon resonance modulation, heterojunction construction, and TME modulation, makes tremendous achievements. With these advances, the remarkable developments of Ti‐based sonosensitizers in biomedical applications have been summarized. Despite the remarkable progress has been made, there are still many obstacles and crucial problems, which should be overcome for promoting the clinical transformation process (**Figure** **13**).

(1) The refined and developed synthesis procedures are essential for large‐scale production. To date, the harsh reaction condition (such as liquid exfoliation) and the complex instrumentation limited scaled‐up synthesis. In addition, the proteins and metabolic processes involved in oxidative stress are mostly unexplored,^[^ ^165^ ^]^ which is a major issue to hinder the development and effective utilization of nanomaterials. Mitigating the toxicity of nanomaterials in vivo should be carried out simultaneously with the development of nanomaterials. More effort needs to be put into developing cost‐effective, standardized, as well as mass‐produced nanomaterials, for the improvement of theranostic performance and saving of treatment cost.
(2) The intrinsic mechanism and principles of novel optimized therapies need to be more clearly revealed for the development of sonosensitizers. Engineering nanosonosensitizers with acceptable therapeutic outcomes and performance has been proved challenging due to the difficulties in establishing the link between physiochemical property and sonodynamic performance.^[^ ^90^ ^]^ Only by clarifying these mechanisms can we provide a theoretical and experimental basis for further optimizing performance.
(3) Ti‐based nanomedicine must undergo a thorough and methodical evaluation of its long‐term biosafety and biocompatibility as a requirement for clinical use. Physicochemical characteristics of Ti‐based nanomedicine, including composition, particle size, shape, solubility, surface structure, deformability, degradability, targeting functionality, as well as colloidal stability, are fundamentally important for understanding their pharmacokinetic behaviors. The optimal design of low‐toxicity, smaller size, and high performance of Ti‐based nanosonosensitizers endowed their capacity to concentrate on tumor cells and be expelled, ensuring improved efficiency and less long‐term biosafety concerns. Despite mounting evidence supporting the beneficial effects of material optimization techniques on biosecurity, achieving the clinical transformation of the corresponding nanomedicine will inevitably require further understanding and revealing of biological effects as well as a systematic review of biosafety and metabolic processes.
(4) Efforts should be devoted to the development of standardized evaluation methods, so that the results from each laboratory are comparable. For instance, standardized ROS productivity indicators can be evaluated after comparison with the same TiO_2_ nanosensitizer.^[^ ^61b^ ^]^ Only by systematically comparing each study can we select the most effective nanoplatform and direct future therapeutic applications.
(5) Last but not least, interaction and collaboration amongst various background researchers are highly necessary for nanomedicine to succeed as a medical‐engineering interdisciplinary field. Considering that the exploration of novel and efficient sonosensitizers is still in the infant stages, we are supposed to integrate material science, physics, biology, and chemistry to make more comprehensive considerations before designing nanosonosensitizers to fight against various diseases. We can pinpoint the precise chemical composition of Ti‐based nanoplatforms based on the characteristics of the therapeutic and diagnostic process. For instance, we can add paramagnetic transition‐metal component(s) to the nanosonosensitizers to enable MR imaging and direct the treatment process and exploit more opportunities in future research on Ti‐based nanosonosensitizers.

Many optimization efforts are increasingly addressing the inherent constraints of Ti‐based nanosensitizers. As elucidated above, a deeper comprehension of the fundamental ideas behind creating and using sonosensitizers is essential.^[^ ^166^ ^]^ With the continuous advancements in bio‐nanotechnologies, it is strongly anticipated that the sonocatalytic optimization of titanium‐based therapeutic nanomedicine would reach significant successes in the nanomedical area.

## Conflict of Interest

The authors declare no conflict of interest.
